# Dissecting endometrial cancer complexity in response to standard and targeted therapies

**DOI:** 10.1038/s41419-025-08051-8

**Published:** 2025-11-28

**Authors:** Sebastiano Vaccarella, Valentina Bruno, Giulia Orlandi, Daniela Angela Covino, Carlotta Frascolla, Claudio Pulito, Riccardo Vizza, Giulia Urbani, Matteo Allegretti, Valentina De Pascale, Frauke Goeman, Ludovica Ciuffreda, Brindusa Ana Maria Arteni, Simona Di Martino, Andrea Sacconi, Emanuela Mancini, Anna Bagnato, Ermelinda Baiocco, Ramy Kajal, Yaron Vinik, Sima Lev, Maurizio Fanciulli, Antonello Vidiri, Mariantonia Carosi, Sabrina Strano, Sara Donzelli, Enrico Vizza, Giovanni Blandino

**Affiliations:** 1 Translational Oncology Research Unit, IRCCS Regina Elena National Cancer Institute, Rome, Italy; 2 Gynecologic Oncology Unit, Department of Experimental Clinical Oncology, IRCCS Regina Elena National Cancer Institute, Rome, Italy; 3 Microbiology and Virology Unit, IRCCS San Gallicano Dermatological Institute, Rome, Italy; 4 SAFU Unit, IRCCS Regina Elena National Cancer Institute, Rome, Italy; 5 Pathology Unit, Tissue Biobank IRCCS Regina Elena National Cancer Institute, Istituti Fisioterapici, Ospitalieri IFO, Rome, Italy; 6 Biostatistics and Bioinformatics Unit, Clinical Trial Center, IRCCS Regina Elena National Cancer Institute, Rome, Italy; 7 Unit of Preclinical Models and New Therapeutic Agents, IRCCS-Regina Elena National Cancer Institute, Rome, Italy; 8 Radiology and Diagnostic Imaging, IRCCS Regina Elena National Cancer Institute, Rome, Italy; 9 Molecular Cell Biology Department, Weizmann Institute of Science, Rehovot, Israel; 10 Pathology Unit, IRCCS, Regina Elena National Cancer Institute, Rome, Italy

**Keywords:** Endometrial cancer, Translational research

## Abstract

Endometrial cancer (EC) is one of the most common gynecologic malignancies amongst women worldwide. Its incidence and mortality rates have been increasing in the last decade. In the present work, we built a patient EC-derived organoid (PDOs) platform that faithfully recapitulated tumor phenotype, genomic alterations, and expression profiles of matched-primary cancer tissues. Interestingly, we found that the response of EC-derived PDOs to both standard therapy and a wide range of targeted drugs accordingly to their specific druggable genetic alterations was congruent with that of the originating patients. We also isolated and genomically characterized matched-PDO stromal cells, specifically cancer-associated fibroblasts (CAFs). Unlike PDOs matched CAFs were poorly responsive and underwent to pro-inflammatory senescence upon treatment with standard therapy. Collectively, our findings established a EC-PDOs preclinical platform which allows assessing the therapeutic response of tumor and surrounding tumor microenvironment cellular landscape.

## Introduction

Endometrial cancer (EC) ranks as the sixth most common cancer in women, with 417,000 new diagnoses made globally in 2020 [1, 2]. The overall incidence has surged by 132% in the past three decades, reflecting a rise in the prevalence of risk factors; in particular, obesity and an ageing population. Although diagnoses have increased across all age groups, there has been a doubling in cases among women under 40 years old, who now account for 4.2% of all low-grade EC diagnosed in the United States [3], with a huge social impact in terms of fertility: about 70% of these young patients are nulliparous at the time of diagnosis [4]. Historically, EC was classified into two clinicopathological subtypes based on estrogen expression. Type I, which is estrogen-dependent and associated with low grade cells, it is also associated with PTEN, KRAS, CTNNB1, and PIK3CA genetic alterations. Patients with this type of cancer have a good prognosis, with an overall 5-year survival rate of 85%. On the other hand, Type II is considered the most aggressive EC, characterized by a poor prognosis, with a high risk of metastasis and relapse. This subtype is characterized by the occurrence of TP53 mutation and oncogenes amplification and the lack of estrogen receptors’ expression [5]. Recently, the Cancer Genome Atlas (TCGA) suggested a new set of criteria for EC classification based on genomic alterations that categorizes endometrial carcinoma into four distinct prognostic molecular subgroups: polymerase ε (POLE) ultra-mutated, microsatellite instability hyper-mutated, copy-number low and copy-number high [6]. The first EC treatment is surgery and the adjuvant treatment is currently based on radiotherapy and standard chemotherapy, characterized by carboplatin and taxolo [7]. To be able to use a patient preclinical model is therefore becoming more and more of interest, since the potential impact in EC research advances could turn out to be huge for patient’s management and treatment in precision oncology area. Patient-derived organoids (PDOs) are self-organizing three-dimensional culture that maintain consistent and phenotypic features of native tumor tissue. Recently, emerging studies have highlighted the value of PDOs systems for analyzing histopathological and molecular heterogeneity of different tumors, including EC [8, 9]. Additionally, organoids can also be used to assess drug response to chemoradiotherapy, target therapy and immunotherapy, to identify responding and non-responding patients and to guide the personalized treatment appropriate for each patient. Tumor microenvironment (TME) is an important promoter of tumor development affecting proliferation, immune evasion, relapse and migration [10]. The TME is a highly variegated network of cancerous and stromal cells, fibroblasts, immune component, collagen, MMP, cytokines, chemokine and non-cancerous cells. Activated fibroblasts, specifically Cancer-Associated Fibroblasts (CAFs), characterized by Alpha-Smooth Muscle Actin (αSMA), Vimentin and Fibroblast Activation Protein α (FAP) biomarkers, are the most abundant component of TME. They are involved in extracellular matrix (ECM) remodeling, in epithelial-to-mesenchymal transition (EMT), in cancer occurrence processes and therapeutic resistance. Consequently, CAFs are assumed to play a prominent role in EC even in the absence of conclusive evidence [11–13]. In the present study, we aimed to develop a 3D culture system of primary endometrial cancer, which faithfully recapitulates histo-pathologically and molecularly their matched primary tumors and to investigate the crosstalk of organoids and patient-matched CAFs, focusing on cancer proliferation and drug chemosensitivity.

## Material and methods

### Human tissue

Fresh tissues were obtained from patients with malignant endometrial diseases, treated in IRCCS-IRE Regina Elena National Cancer Institute. The Ethical Research Committee approved this study. All patients gave written informed consent.

### Blood processing method

A total of 6 mL blood was collected from each participant in ethylenediaminetetraacetic acid (EDTA) tubes. Blood samples were processed, with one milliliter cryopreserved within 2 h after collection. The remaining fresh samples were used for PBMC isolation.

### Isolation of PBMC by Ficoll density gradient centrifugation

PBMC were isolated from peripheral blood using Ficoll (CL5020, Lympholyte, Euroclone). Briefly, the remaining 5 ml blood was diluted with an equal volume of phosphate-buffered saline, pH 7.4 (PBS). 12.5 ml of diluted blood was layered over 25 ml of the Ficoll. Gradients were centrifuged at 800 × *g* for 20 min at room temperature in a swinging-bucket rotor without the brake applied. The PBMC interface was carefully removed by pipetting and washed with PBS by centrifugation at 250 × *g* for 10 min. PBMC pellets were suspended in 3 ml of ammonium-chloride-potassium lysing buffer (07850, STEMCELL Technologies) and incubated for 5 min on ice with gentle mixing to lyse contaminating red blood cells (RBC). This, followed by a wash with PBS, was centrifuged at 1000 × *g* for 10 min and stored at −80 °C.

### Establishment of organoids culture

Collected samples were maintained in MACS Tissue Storage Solution (130-100-008, Miltenyi Biotec) supplemented with 100 U/ml penicillin, 100 μg/ml streptomycin and 100 μg/ml antimycotic for a maximum of 24 h at 4 °C. Subsequently, the samples were washed twice in PBS, mechanically minced in a petri dish, and the small pieces were transferred to gentleMACS C Tubes (130-093-237, Miltenyi Biotec) or storage at −80 °C tissue into the cryotubes with 1 ml of MACS Freezing solution for each cryovial (130-129-552, Miltenyi Biotec). The single-cell suspensions were obtained using Tumor Dissociation Kit (130-095-929, Miltenyi Biotec), according to the manufacturer’s instructions. The obtained cellular suspension was then filter with a 70 µm strainer (130-098-462, Miltenyi Biotec). Dissociated cell clusters were spun down at 1200 rpm for 5 min, washed once with PBS, and spun down again at 1200 rpm for 5 min. If the pellet showed a visible red color, erythrolysis was performed with Ammonium Chloride Solution (07850, STEMCELL Technologies) before the washing step. Dissociated cell clusters were resuspended in cold Matrigel (356231, Corning) and seeded in a prewarmed 24-well plate at density of 6 × 10^5^ cells per 30 µl drops. The drops were solidified in a 37 °C and 5% CO_2_ incubator for 30 min, and then 500 µl organoid culture medium (Advanced DMEM/F-12 (12634010, Gibco), 200 mM GlutaMAX (35050038, Gibco), 1 mM HEPES (15630080, Gibco), R-Spondin 1 (HZ-1328-1000UG, HumanKine), 100 ng/mL Noggin (HZ-1118-1000UG, HumanKine), 1.25 mM N-Acetylcysteine (A9165, Sigma Aldrich), 5 mM Nicotinamide (N0636, Sigma Aldrich), 1X N2 supplement (17502001, Invitrogen), 1X B27 supplement (17504001, Invitrogen), 1% Chemically defined Lipid concentrate (11905031, Gibco), 250 nM A83-01 (S7692-25MG, HumanKine), 50 ng/mL EGF (HZ-1326-1000UG, HumanKine), 40 ng/mL IGF-1 (HZ-1322-1000UG, HumanKine), 20 ng/mL HGF (HZ-1084-100UG, HumanKine), 5 ng/mL IL-6 (HZ-1019-100UG, HumanKine), 100 nM SB202190 (S7067, Sigma Aldrich), 10 nM 17 β-estradiol (E8875, Sigma Aldrich), 10 μM Y-27632 (HY-10583-10MG, HumanKine) was added to each well and refreshed every 2–3 days. The organoids were passaged every 5–9 days, depending on proliferation rate.

### Cancer-associated fibroblast isolation and culture

Cancer-associated fibroblasts were isolated from organoids droplets after 3–4 days. When the PDOs were passaged, CAFs were found on the bottom of droplets. After first washing twice in PBS and cell trypsinization, they were transferred to a flask or dish. CAFs were cultured in RPMI 1640 medium supplemented with 10% fetal bovine serum (Gibco), 200 mM GlutaMAX (Gibco), 100 U/ml penicillin, 100 μg/ml streptomycin and 100 μg/ml antimycotic, passaged at approximately 80% confluence every 4–5 days and verified using immunostaining for αSMA, FAP and Vimentin as positive controls and EpCAM, CD45, and CD31 as negative controls.

### Immunohistochemical analysis

Organoids were resuspended with 10 mL of Cell Recovery Solution (Corning, 354270) and incubated on ice for 1 h. Then they were centrifuged at 500 × *g* for 5 min at 4 °C. The supernatant was removed and the pellet was resuspended in 10 mL of Thin Prep Solution. Afterwards, samples were processed by The Celllient^TM^ Automated Cell Block System (Hologic Corporation, Marlborough, MA) which is fully automated and creates a paraffin-embedded cell block using isopropanol for dehydratation and xylene for clarification. Cell blocks were cut at 3 μm using a microtome LEICA SM 2000R (Advanced Research Systems Inc., Macungie, PA) and mounted onto slides. The slides were dewaxed in xylene and rehydrated through a series of graded ethanol solutions and stained with Gill’s Hematolylin (Bio-optica, Milan) and Eosin (Bio-optica, Milan).

The slides were incubated with the following primary antibodies: estrogen receptor (clone 6F11, LEICA), progesterone receptor (clone 16, LEICA), TP53 (clone DO7, LEICA), vimentin (clone V9, LEICA), CKAE1/AE3 (clone AE1/AE3, LEICA), αSMA (clone ASM1, LEICA) in an automated immunostainer (Bond-III, Leica, Biosystems, Italy), according to the manufacturer’s instructions. A citrate buffer, pH 6 or pH 8, was used to unmask the antigens in each case. Images were obtained at magnification 20x by using Aperio Image Scope system equipped with a Digital Image Capture software. The evaluation was based on the percentage of positive cells.

### Immunofluorescence

For immunofluorescence assay, 1 × 10^3^ cells/well were seeded in culture media onto 96-wells Pheno Plate (6055300, Revvity). Cells were fixed with 4% paraformaldehyde in PBS for 15 min at room temperature and then permeabilized with 0.1% Triton X-100 in PBS for 10 minutes. After blocking with 2% BSA/PBS at room temperature slides were incubated in 0.1% BSA/PBS overnight at +4° with primary antibodies: mouse monoclonal anti-EpCAM (#2929, Cell Signaling), rabbit monoclonal anti-p21 (#2947, Cell Signaling), rabbit anti-αSMA antibody (#19245, Cell Signaling), mouse monoclonal anti-vimentin (sc-66001, Santa Cruz Biotechnology), rabbit polyclonal anti-p16 (sc-467, Santa Cruz Biotechnology), mouse monoclonal anti-p53 (sc-126, Santa Cruz Biotechnology) according to the manufacturer’s instructions. Secondary antibody used were Alexa Fluor 488 (1:500; Thermo Fisher Scientific); Alexa Fluor 647 (1:500; Thermo Fisher Scientific). Nuclei were stained with DAPI (Thermo Fisher Scientific).

Images were obtained at air objective 10x and water objective ×20 magnification by using Opera Phenix Plus high throughput microplate confocal imager (Revvity, Massachusetts, USA).

### 3D immunofluorescence

For 3D immunofluorescence assay, PDOs were cultured in 3 μl of Matrigel and seeded into 96-well Pheno Plate (6055300, Revvity), using the Corning Matribot bioprinter. PDOs were fixed with 4% paraformaldehyde in PBS for 30 min at room temperature. After blocking with 2% BSA/PBS at room temperature, the wells were incubated in 0.1% BSA/PBS for 2 days at +4° with mouse monoclonal anti-EpCAM (#2929, Cell Signaling), according to the manufacturer’s instructions. Then gently remove the primary antibody solution, PDOs were permeabilized with 0.1% Triton X-100 in PBS for 15 min. After twice washing with PBS, blocking with 2% BSA/PBS at room temperature slides were incubated in 0.1% BSA/PBS overnight at +4° with rabbit anti-aSMA antibody (#19245, Cell Signaling), mouse monoclonal anti-vimentin (sc-66001, Santa Cruz Biotechnology), according to the manufacturer’s instructions. Secondary antibody used were incubated in 0.1% BSA/PBS for one day at +4° with Alexa Fluor 488 (1:500; Thermo Fisher Scientific); Alexa Fluor 647 (1:500; Thermo Fisher Scientific). Nuclei were stained with DAPI (Thermo Fisher Scientific).

Images were obtained at air objective 10x and water objective ×20 magnification by using Opera Phenix Plus high throughput microplate confocal imager (Revvity, Massachusetts, USA).

### Monoclonal antibodies

Monoclonal primary antibodies applied for flow cytometry analyses were purchased from BD (CD31, CD45, CD326), Life Technologies (vimentin), Novus (αSMA) and R&D systems (FAP), respectively. Working dilutions were determined by titration and compared to their relative control isotype to define optimal thresholding.

### Flow cytometry analyses

CAFs were harvested from culturing plates by TrypLE Express (Gibco™, Life Technologies) and washed twice with ice-cold PBS/0.5% BSA. Then, live&dead staining was performed for 30 min at room temperature. Cells were centrifuged, washed once and labeled with a cocktail of monoclonal antibodies to surface markers (e.g., CD31, CD45, CD326, FAP) for 30 min at 4 °C. After centrifugation, cell pellets were fixed by PFA 4% for 15 min at room temperature and permeabilized with PBS/0.1% Triton X-100. Then, monoclonal antibodies to intracellular markers (e.g., αSMA, vimentin) were incubated for 30 min at room temperature. Fluorescent signals were measured by a 4-laser Cytoflex S flow cytometer (Beckman Coulter, Indianapolis IN, USA). A minimum of 10,000 events were collected and further analyzed with FlowJo v10.1 (FlowJo LLC, Ashland, OR, USA). Isotype controls were used to properly set the compensation matrix and reduce non-specific labeling.

### Whole exome sequencing

DNA was extracted from frozen patient tissue, organoids and CAFs using AllPrep DNA/RNA/miRNA Universal Kit (80224, QIAGEN) according to the manufacturer’s instructions. Genomic DNA was extracted from frozen frozen matched-blood or matched-PBMC using kit (51104, QIAGEN) according to the manufacturer’s instructions. Genomic DNA was quantified using Qubit dsDNA BR Assay Kit (Invitrogen, Carlsbad, CA, USA). Quality was determined (DIN range from 1 to 10) on 4200 TapeStation using Genomic DNA screenTape assay (Agilent Technologies, Santa Clara, USA).

Pre-enrichment libraries were performed using 100 ng of DNA according to Library Preparation EF 2.0 with Enzymatic Fragmentation and the Twist Universal Adapter System (Twist Bioscience, San Francisco, CA, USA) according to the manufacturer’s instructions. Exome hybridization conducted using a Twist Comprehensive Exome kit (Twist Bioscience, CA) according to the manufacturer’s protocol. This protocol provides coverage for more than 99% of protein-coding genes. The quality of the libraries was assessed using the Agilent 4200 TapeStation system (High Sensitivity D1000 ScreenTape assay), while their quantity was measured by qPCR. The exome library was sequenced on the Illumina NovaSeq 6000 (Illumina, San Diego, CA, USA) platform with 100 bp paired-end reads.

### RNA sequencing

RNA was extracted from fresh frozen tissues, organoids and CAFs using AllPrep DNA/RNA/miRNA Universal Kit (80224, QIAGEN) according to the manufacturer’s instructions. The quality of the RNA has been controlled on a Bioanalyzer with the RNA 6000 Nano kit (Agilent Technologies, Santa Clara, CA, USA). The libraries for the RNA-Sequencing have been prepared using the TruSeq RNA Exome kit following the manufacturer’s instructions. The resulting libraries have been quality controlled on a Bioanalyzer with the High Sensitivity DNA Kit (Agilent Technologies, Santa Clara, CA, USA) and quantified by qPCR. The sequencing has been performed on a NovaSeq 6000 instrument (Illumina Inc., San Diego, CA, USA), sequencing in paired-end mode 101 bp x 2.

### digital PCR and custom assays

Custom-designed primers and probes will be created based on the specific mutation profiles of the cells, with synthesis provided by Integrated DNA Technologies (IDT). Each probe will be synthesized as an Affinity Plus® Probe and undergo HPLC purification for optimal quality.

The samples were analyzed simultaneously using the Quant Studio™ Absolute Q Digital PCR system (Life Technologies), which employs a chip/plate-based format. Each reaction was prepared in a final volume of 10 μL, consisting of 2 μL of 5x Master Mix, 0.5 μL of 20x TaqMan® probe, and 7.5 μL of the DNA template, before loading onto the dPCR plates. The thermal cycling conditions were as follows: an initial denaturation step of 10 min at 96 °C, followed by 39 cycles of 96 °C for 5 s and 60 °C for 15 s.

Fluorescence threshold values for FAM and VIC were automatically determined by ThermoFisher software, manually reviewed for accuracy, and subsequently applied to the respective DNA samples.

### Cell and organoid treatment

In all, 1 × 10^3^ CAFs were seeded into 96-well plates. 6 × 10^4^ cells for PDOs formation were cultured in 3 μl of Matrigel and seeded into 96-well Pheno Plate (6055300, Revvity), using the Corning Matribot bioprinter. Allowed cells and organoids growth, they were treated for 72 h with following compounds from Selleck Chemicals: Carboplatin (50-200 μM) (S1215), Paclitaxel (50-200 nM) (S1150), Carboplatin-Paclitaxel (50/100/200 μM Carboplatin + 50/100/200 nM Paclitaxel), Gedatolisib (0.1-1-10 μM) (S2628), Buparlisib (0.1-1-10 μM) (S2247), Alpelisib (0.1-1-10 μM) (S2814), Rigosertib (0.1-1-10 μM) (S1362), Volasertib (0.1-1-10 μM) (S2235), Ipatasertib (0.1-1-10 μM) (S2808), Adagrasib (0.1-1-10 μM) (S8884), Olaparib (0.1-1-10 μM) (S1060), Trastuzumab deruxtecan (D4001) or DMSO.

To prepare all the dilution and dispense the compounds, we utilized the Zephyr G3 NGS workstation, a high-precision multichannel liquid handler.

### Viability assay

Cells and PDOs viabilities were assessed using ATPlite luminescence assay (Revvity, Massachusetts, USA) and Live-Dead Cell Viability Assay Kit (Merck Millipore), according to the manufacturer’s instructions. Half maximal inhibitory concentration (IC50) was calculated by software according to the manufacturer’s instructions. To evaluate the viability, we utilized EnSPIRE (Revvity, Massachusetts, USA) and Opera Phenix Plus high throughput microplate confocal imager (Revvity, Massachusetts, USA).

The SD and *p-value* were calculated from three independent experiments (*p < 0.05; **p < 0.001; ***p < 0.0001).

### Senescence assay

SA β-galactosidase hydrolysis was assessed using CellEvent Senescence Green Detection kit (Invitrogen) according to the manufacturer’s instructions.

### Morphological parameters

Percentage of live, area, width, perimeter and roundness dimensions of the CAFs and organoids was assessed by using the Opera Phenix Plus high throughput microplate confocal imager (Revvity, Massachusetts, USA) and calculated by using Harmony High-Content Imaging and Analysis Software.

Opera Phenix Plus achieves higher throughput and richer content. The system uses the Harmony acquisition and image analysis software which is powerful and simple to use for 2D and 3D cultures. To identify PDOs, we filtered the brightfield images by applying texture SER method. Then we trained machine learning software basing on SER Dark properties to identify PDOs population. The obtained population was selected basing on its morphology properties such as area greater than 200 µm² and a roundness greater than 0.8, this allows us to distinguish PDOs from single cells and matrigel background. We then calculated the texture properties of the selected population by applying the SER features method. This allows us to training software in distinguish live to death PDOs [14].

The software also calculates the means of percentage of live (%), area (µm^2^), width (µm), perimeter (µm) and roundness dimensions of each well. PDOs brightfield images showed in the manuscript are representative of the morphological parameters’ mean calculated by Harmony software, illustrated with histograms or heatmaps.

### Cytokine secretion assays

The Biotechne protein simple plex cartridge kit was used for measuring the levels of IL-6 in culture media samples. Specifically, samples were centrifuged at 12,000 rcf in the microcentrifuge for 10 min, 25 μl of the supernatant was, then, diluted in 25 μl (1:1) of sample diluent. The 50 μl of diluted sample was loaded into the simple plex cartridge according to the manufacture’s instruction.

### Bioinformatic analysis

WES data were processed with Sarek version 3.4.0, an nf-core pipeline (https://nf-co.re/sarek) designed for the identification of germline and somatic variants. Gene and variant annotations were performed using the Variant Effect Predictor (VEP), while oncogenic and clinically relevant mutations were identified with the Personal Cancer Genome Reporter (PCGR) tool. Mutation sites were selected based on specific criteria: a minimum of 20 sequencing reads and a minor allele frequency (MAF) of 0.5 or greater. CNVkit v0.3.5 was employed to analyze sequencing coverage and copy number variations, while CNAqc v1.0.0 (10.1101/2021.02.13.429885) was used to visualize the loss of heterozygosity (LOH) status.

RNA-seq data were analyzed using the “rnaseq” version 3.9 pipeline integrated into the nf-core platform (https://nfco.re/rnaseq) with default parameters (10.5281/zenodo.5550247). Unsupervised hierarchical clustering of the pathways was performed by calculating a score with the ssGSEA function in R. ssGSEA was performed using the ssGSEA module from GenePattern (https://cloud.genepattern.org/gp/pages/login.jsf), including pathways from HALLMARK, REACTOME, and KEGG databases. The ComplexHeatmap R package was used for both unsupervised hierarchical clustering and Oncoprint visualization. Tumor microenvironment (TME) was investigated with Molecular Functional Portrait (10.1016/j.ccell.2021.04.014) to classify the samples into one of the TME subtypes and comparing matched tissues and PDOs.

To identify the four subgroups immune-enriched fibrotic (IE/F), immune-enriched (IE), fibrotic (F), and depleted (D) was used single-sample Gene Set Enrichment Analysis (ssGSEA) based on a set of 29 functional genes. This classification enabled the grouping of tumor samples according to their immune and fibrotic characteristics.

Pearson’s correlation test was executed in R to assess the relationship between tissue samples and the corresponding PDOs.

## Results

### Establishment and immunohistochemical validations of organoids derived from different EC subtypes

**Fig. 1:**
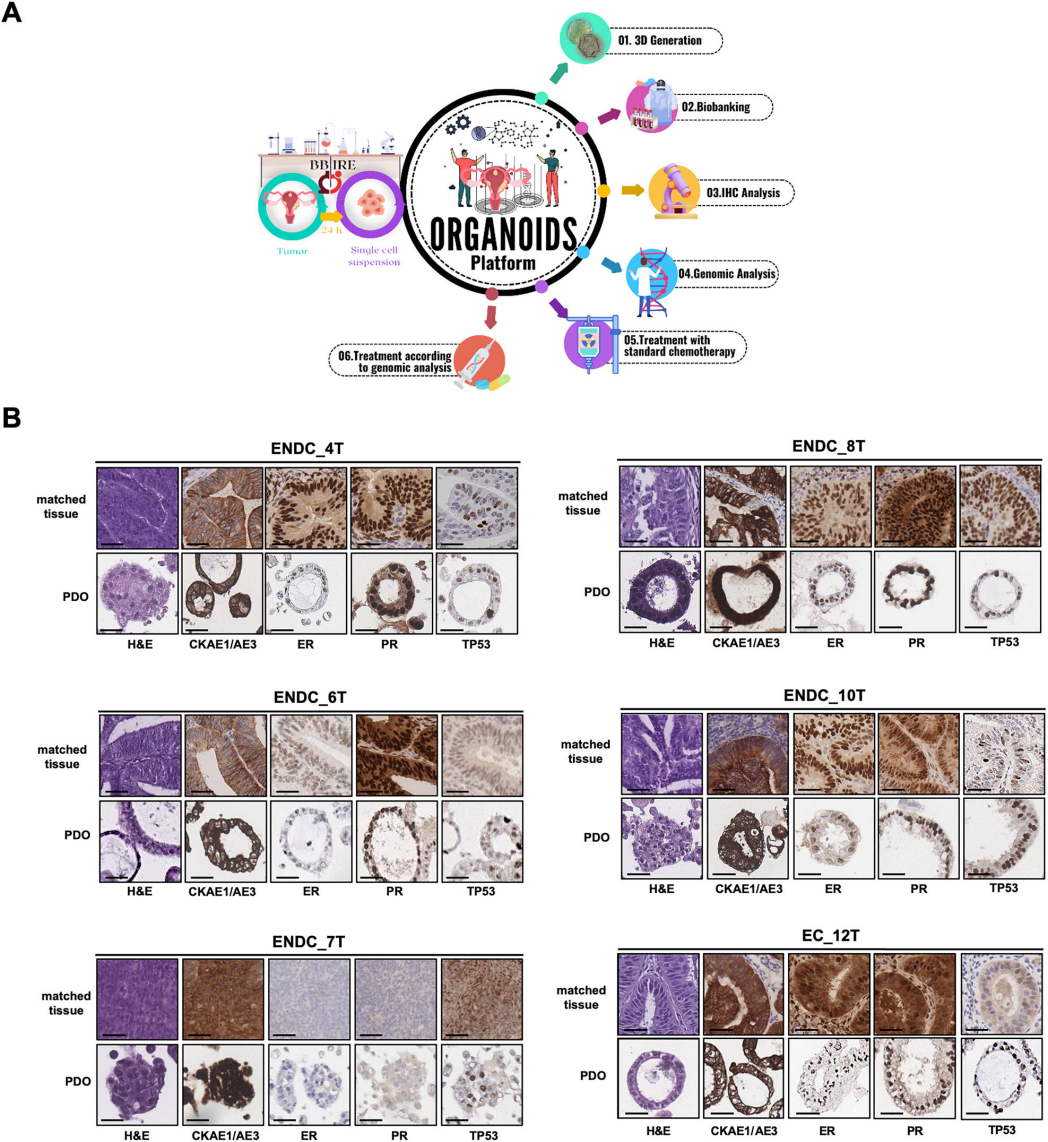
Establishment of EC PDOs. **A** Schematic overview of organoid establishment and platform validation. **B** Comparative histological analysis of endometrial cancer (EC) matched-tissues and patient derived organoids culture (PDOs). (H&E: hematoxylin and eosin; CKAE1/AE3: Cytokeratin, Clones AE1/AE3; ER: estrogen receptor; PR: progesterone receptor; TP53). Scale Bar: 50 µm.

To generate PDOs consecutive enrolment of EC patients referred to the Italian National Cancer Regina Elena Institute was approved from Institutional Ethical Board (*RS1777/22(2764)*). To date 136 untreated patients affected by EC were consecutively enrolled in the study (Fig. S1A). The first part of this study was designed as a feasibility study, in which we demonstrated the ability to generate 10 PDOs from consecutively enrolled EC patients. PDO cultures were successfully established for a long-term expansion and cryopreserved for biobanking from 24 out 30 EC patients with 80% success rate (Fig. S1B). Clinical information about patients and corresponding PDOs are reported in Supplementary Tables S1 and S2. Figure 1A illustrates the workflow for PDOs platform generation, biobanking, genomic validation, and treatment with standard and targeted therapies. After surgery, a small portion of the tumor was collected and processed. One piece of the sample was stored at −80 °C for planned genomic evaluation, while the remaining part of the tumor was processed for organoid generation. PDOs, maintained in the optimal conditions described in the Materials and Methods section, developed within 5–9 days and were expanded at a passage ratio of 1:2 for up to 2 months. EC subtype classification is primarily based on H&E staining and immune-histological (IHC) analysis of hormone receptors (estrogen (ER) and progesterone (PR) receptors), CK-AE1/AE3 (epithelial cell markers) and TP53. Interestingly, the H&E staining on native tumor and the matched-PDOs revealed that the PDOs typically preserved the histological and cellular architecture, mirroring the specific features of patient’s tumor subtype (Fig. 1B). Additionally, the staining of primary tissue markers was maintained in the respective organoids derived by several endometrial adenocarcinomas of different stages and grades, such as 6T_PDO and 7T_PDO (Fig. 1B). Intriguingly, we observed significant heterogeneity in the size, morphology and structures of EC-PDOs, which appears to correlate with tumor histology. Specifically, organoids derived from EC_6T and EC_10T, which are differentiated endometrial adenocarcinoma (G2), have a cystic structure. In contrast, 7T_PDO derived from a dedifferentiated endometrial adenocarcinoma (G3), displays a solid a structure. Altogether, these findings show that PDOs exhibit remarkable histological similarity with the matched EC tissues.

### EC-PDOs exhibit genomic concordance with matched EC tissues

**Fig. 2:**
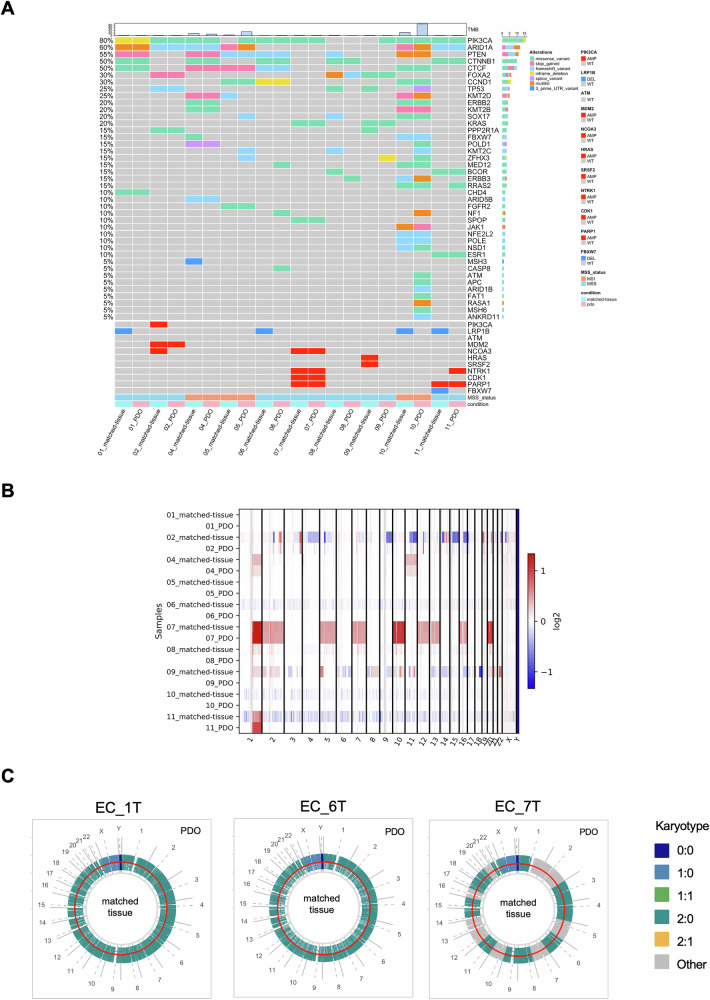
Genomic concordance between PDOs and primary EC tissues. **A** Heatmap of gene mutations and CNA between parental tumor and corresponding PDO in the most frequently mutated genes of EC. Microsatellites status and TMB are reported on the bottom and on the top, respectively. **B** Heatmap of CNA in each chromosome of all EC tissue and PDO samples. Copy number is transformed as log2 ratios per gene (red, gains; blue, losses). **C** Overall karyotype profiles between PDOs (outside the red circle) and matched-tissues (inside the red circle) for three selected cases (Pt#1 T left, Pt#6 T middle, Pt#7 T right).

Whole-Exome Sequencing of both matched-tissues and PDOs was performed to assess the potential genomic similarity between organoids and their corresponding tissues. First, we distinguished between germline and somatic mutations by filtering DNA from tumors and organoids using blood or peripheral blood mononuclear cells (PBMCs), when available. We then compared the 41 most frequently mutated genes in EC, according to TCGA database [15], between the primary tumor and corresponding organoid. These analyses revealed that most mutations present in the primary tumors were retained in PDOs (Fig. 2A). Additionally, we evaluated the Variant Allele Frequency (VAF) of various mutated genes, such as PIK3CA (*p.H1047R, p.C420R, p.E542Q, p.R88Q, p.F83S*), KRAS (*p.G12V, p.G12D*), TP53 (*p.P72R, p.H193L*) AKT3 (*p.E232K*) and MTOR (*p.E2419K*), using both Next Generation Sequencing (NGS) and digital PCR (dPCR) for subsequent validation. Pearson correlation analysis showed that the VAF mutation rates of main mutated genes in the primary tumors were significantly maintained in the corresponding organoids in both assays (*R* = 0.9658, *p* < 0.0001 and *R* = 0.79, *p* = 0.0001, respectively) (Fig. S2A and S2B and Supplementary Table S3). Hierarchical-clustering analysis comparing the genomic mutation in tissue samples with publicly available EC cohorts from The Cancer Genome Atlas (TCGA), consisting of 512 EC samples, confirmed that the samples used in this study are representative of the overall population of primary EC (Fig. S2C and S2D). Accordingly, to TCGA dataset we observed PIK3, PTEN, ARID1A and CTNNB1 genes are mutated in 70%, 60%, 60% and 50% of the patients, respectively (Fig. 2A and S2D). Across all sequenced samples, we further investigated the level of Copy Number Alterations (CNA) distribution between matched-tissues and PDOs (Fig. 2B). As seen in Fig. 2C, the LOH distribution of PDOs was very similar to that of tumoral tissues, hence suggesting that organoids faithfully recapitulate the original tumor heterogeneity.

### EC-PDOs resemble gene expression profile of primary-matched EC patients

**Fig. 3:**
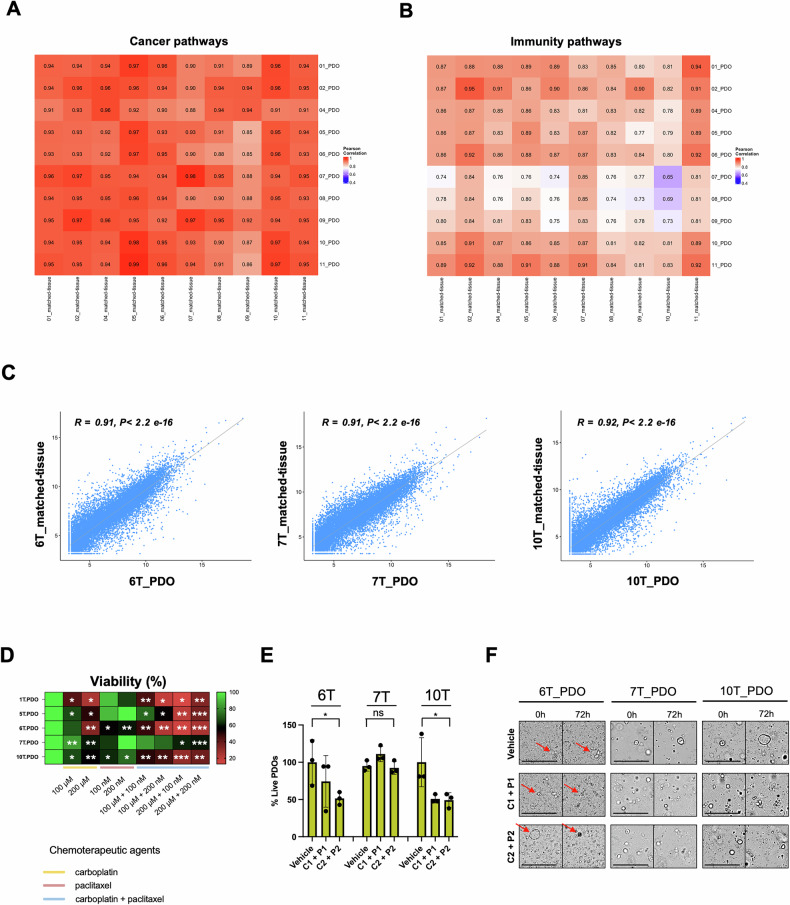
PDOs maintain gene expression profile of matched-tissues and predict response to standard chemotherapy. Correlation heatmaps of cancer (**A**) and immune (**B**) pathways between matched-tissues and PDOs. **C** Pearson correlation analysis between matched-tissue and PDO of three selected cases (Pt#6 T left, Pt#7 T middle, Pt#10 T right). **D** Heatmap of PDOs viability following carboplatin (100–200 µM), paclitaxel (100–200 nM), carboplatin + paclitaxel (100 µM + 100 nM; 100 µM + 200 nM; 200 µM + 100 nM; 200 µM + 200 nM) treatment for 72 h, evaluated by ATPlite luminescence assay. The SD and *p-value* is calculated from three independent experiments (*p < 0.05; **p < 0.001; ***p < 0.0001). **E** Histograms showing the percentage of live PDOs between standard chemotherapy-sensitive (6T_PDO and 10T_PDO) and standard chemotherapy-resistant (7T_PDO) organoid samples after 72 h of treatment. Column graphs show colony count and *p-value* from three independent experiments (*p < 0.05; **p < 0.001; ***p < 0.0001). **F** Representative PDOs brightfield images of the morphological parameters’ mean calculated by Harmony software, illustrated with histograms. Brightfield images showing the morphological differences between standard chemotherapy-sensitive (6T_PDO and 10T_PDO) and standard chemotherapy-resistant (7T_PDO) organoid samples after 72 h of treatment. The red arrows indicate structurally disrupted organoids. Scale bar: 200 µm.

To further explore the similarity between organoids and their corresponding tumor tissues, bulk RNA-sequencing analysis was performed in samples used for WES analysis. Specifically, correlation heatmaps based on single sample Gene Set Enrichment Analysis (ssGSEA) were conducted, focusing on cancer-related (Fig. 3A) and immunity-pathways (Fig. 3B). We observed a high degree of similarity in gene expression between PDOs and their corresponding tissues for cancer related pathways, particularly across key oncogenic signatures (Fig. 3A and Fig. S3A upper panels). Unlike cancer-related pathways genes related to immune and vascular components resulted slightly downregulated in PDOs compared to their matched-tissues (Fig. 3B and Fig. S3A lower panels). Interestingly, the expression of genes associated with epithelial-to-mesenchymal transition (EMT) and stromal cells, including fibroblasts, was very similar between PDOs and their corresponding tumors (Fig. S3A upper panel). As shown in Fig. S3B, to identify the four subgroups immune-enriched fibrotic (IE/F), immune-enriched (IE), fibrotic (F), and depleted (D) was used ssGSEA based on a set of 29 functional genes. This classification reveals that 80% of the evaluated samples maintained the same subtype as their corresponding primary tissue [16]. Additionally, Pearson’s correlation coefficient, calculated for approximately 13.000 genes that were most highly expressed in both tumor and organoid samples, further confirmed the strong similarity of PDOs with their corresponding primary matched-tissues (Fig. 3C and S3C). In aggregate, these findings show that EC_PDOs resemble similar gene expression patterns and altered pathways when compared to matched primary tumor tissues.

### EC_PDOs response to standard therapy for EC patients

To evaluate EC organoids as reliable pre-clinical disease models, we tested the effect of standard therapies on their viability using a metabolic ATPlite luminescence assay and morphological parameters, calculated by EnSPIRE and Opera Phenix Plus High-Content Screening System. First, we developed an automated platform for computation-based drug screening using organoids. To enhance the reproducibility across different wells, we optimized the process by dissociating the organoids into single-cell suspensions, which were then seeded into 96-well plates using the Matribot 3D Bioprinter (Corning). After organoid growth, PDOs were treated with clinically relevant doses of carboplatin (100–200 µM), paclitaxel (100–200 nM) and carboplatin + paclitaxel (100 µM + 100 nM, 100 µM + 200 nM, 200 µM + 100 nM, 200 µM + 200 nM) for 72 h (Fig. S3D and Supplementary Table S4). Most of the PDOs, such as 1 T, 2 T, 6 T, 8 T and 10 T PDOs, showed high sensibility to combination chemotherapy treatment, exhibiting significant reduction in cell viability at low drug concentrations. On the other hand, 7 T and 9 T PDOs showed minimal response to treatment, consistent with their tumor diagnosis and hystotype (Fig. 3D and S3E). Specifically, 1 T, 4 T, 5 T, 6 T and 10 T tumors are classified as differentiated endometrial adenocarcinoma (G2) with good prognosis. In contrast, 7 T tumor is a dedifferentiated endometrial adenocarcinoma (G3) and 9 T tumor is an endometrial carcinosarcoma. It has been reported that both dedifferentiated adenocarcinoma and endometrial carcinosarcoma subtypes are rare, aggressive subtypes associated with poor prognosis and diminished response to standard chemotherapy [17, 18]. Based on viability results and PDOs available, we focused on two chemotherapy-sensitive organoids (6 T and 10 T PDOs) and one chemotherapy-resistant organoid (7T_PDO), to evaluate the percentage of live PDOs using the Opera Phenix’s machine learning.

The Opera Phenix Plus high-content imaging system is designed for high-throughput, high-content imaging assays, including phenotypic screening and viability assays. It utilizes morphological parameters such as roundness, area, perimeter, and width. By analyzing these parameters across different fields and stacks for each PDO, the software differentiates live from dead PDOs, delivering 3D-related phenotypic readouts. Intriguingly, we obtained results that were consistent with those from the ATPlite luminescence assay (Fig. 3E, F and Supplementary Table S5).

In aggregate our findings show that EC-PDOs platform is a valuable pre-clinical tool to test the efficacy of standard chemotherapy.

### EC-PDOs as a tool to test efficacy of targeted therapy

**Fig. 4:**
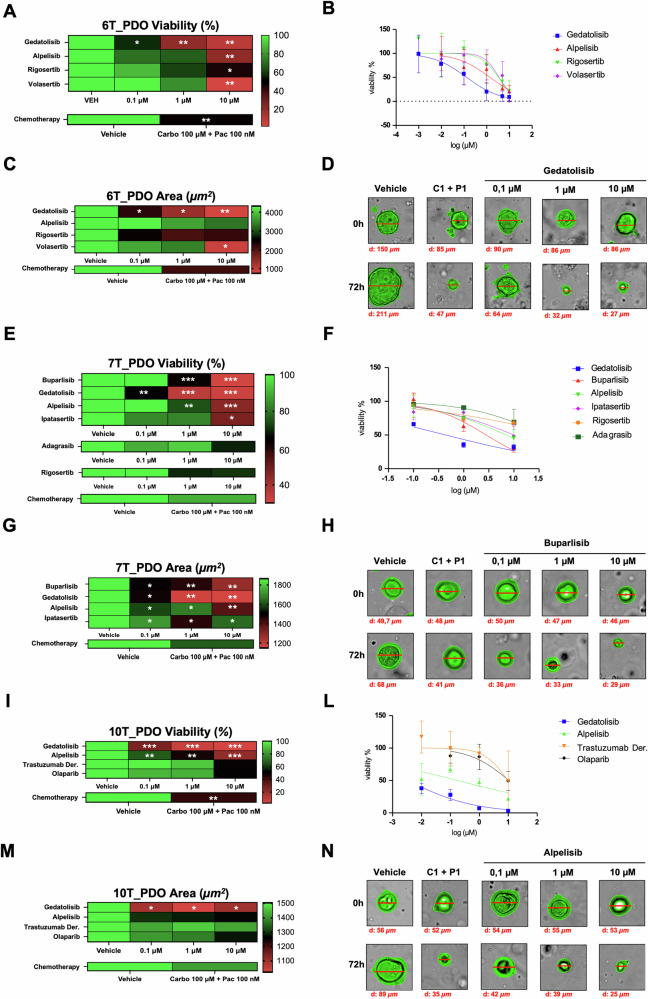
Drug screening of EC PDOs to target therapy agents according to genomic alteration by WES analysis compared with standard chemotherapy. **A**, **E**, **I** Heatmaps of PDOs viability of 6T_PDO (A), 7T_PDO (E) and 10T_PDO (I) following gedatolisib (0.1-1-10 µM), alpelisib (0.1-1-10 µM), rigosertib (0.1-1-10 µM), volasertib (0.1-1-10 µM), buparlisib (0.1-1-10 µM), ipatasertib (0.1-1-10 µM), adagrasib (0.1-1-10 µM), olaparib (0.1-1-10 µM), Trastuzumab deruxtecan (0.1-1-10 µM) and carboplatin (100 µM) + paclitaxel (100 nM) treatment for 72 h, evaluated by ATPlite luminescence assay. The *p-value* is calculated from three independent experiments (*p < 0.05; **p < 0.001; ***p < 0.0001). **B**, **F**, **L** Representative dose-response curve for adagrasib, alpelisib, buparlisib, gedatolisib, rigosertib, ipatasertib, volasertib, olaparib and trastuzumab deruxtecan evaluated by ATPlite luminescence assay. **C**, **G**, **M** Each curve depicts the average of three biological replicates. Heatmaps of 6T_PDO (**C**), 7T_PDO (**G**) and 10T_PDO (**M**) area following gedatolisib (0.1-1-10 µM), alpelisib (0.1-1-10 µM), rigosertib (0.1-1-10 µM), volasertib (0.1-1-10 µM), buparlisib (0.1-1-10 µM), ipatasertib (0.1-1-10 µM), adagrasib (0.1-1-10 µM), olaparib (0.1-1-10 µM), trastuzumab deruxtecan (0.1-1-10 µM) and carboplatin (100 µM) + paclitaxel (100 nM) treatment for 72 h, evaluated by Opera Phenix Plus high throughput microplate confocal imager. The *p-value* is calculated from three independent experiments (*p < 0.05; **p < 0.001; ***p < 0.0001). **D**, **H**, **N** Representative PDOs brightfield images of the morphological parameters’ mean calculated by Harmony software, illustrated with heatmaps. Brightfield images showing the morphological differences of 6T_PDO (**D**), 7T_PDO (**H**) and 10T_PDO (**N**) between 0 h (up) and 72 h (down) of treatment. The red lines indicate the representative diameter (µm) of PDOs.

Drug sensitivity testing using 3D preclinical models is a valuable, efficient, and rapid approach for predicting therapeutic efficacy in individual patients. Based on the robust similarity between the organoid and the matched-tissue, we assessed the feasibility of using EC organoid platform to test the efficacy of targeted therapies guided by WES results. Accordingly, we treated three representative organoid cultures, 6T_PDO, 7T_PDO, and 10T_PDO, with specific molecular drugs at clinically relevant doses and compared their response to those obtained from chemotherapy treatments [19–27]. The 6T_PDO sample harbors PIK3CA *(p.C420R*) and PLK1 (*p.R364Q*) mutations. Accordingly, the 6T_PDO was treated either with alpelisib, a selective inhibitor of the α-isoform of the catalytic subunit of PI3K, gedatolisib, a dual inhibitor of all the isoforms of the catalytic subunit of PIK3 and mTOR, rigosertib, which targets both PLK and PI3K signaling, and volasertib, a specific inhibitor of the PLK1 ATP-binding pocket. The viability of the 6T_PDO sample was significantly reduced at 0.1 µM of gedatolisib and 10 µM of the other molecular drugs compared to dose of chemotherapy (carboplatin 100 µM plus paclitaxel 100 nM) (Fig. 4A, B). Interestingly, the half-maximal inhibitory concentration (IC50) of 6T_PDO treated with gedatolisib, alpelisib, and rigosertib reflects the value of plasmatic concentration of targeted compounds (Fig. S4A, S4B and Supplementary Table S6). This result was further supported by a more pronounced reduction in the organoid area, the width and the perimeter following targeted therapy, compared to standard chemotherapy treatment (Fig. 4C, D, S4C and S4D). Similarly, the 7T_PDO sample harbors a mutation in the alpha helic domain of PIK3CA, (*p.E542Q*), previously found in EC [28], and a mutation in protein kinase domain of AKT3 (*p.E232K*). Consequently, we tested the efficacy of PIK3CA inhibitors such as alpelisib, gedatolisib, and buparlisib, a pan-class PIK3 inhibitor, and ipatasertib, an AKT signaling inhibitor, on 7T_PDO. As shown in Fig. 4E–H, S4E and S4F, target therapies tailored to these specific mutations proved to be more effective than standard chemotherapy. To further investigate the platform’s specificity for targeted drug screening and gain insights into the mechanisms of targeted therapies, we also treated the 7T_PDO sample, which carries KRAS *p.G12D* mutation, with adagrasib, a KRAS inhibitor specifically used for the targeting of *p.G12C*-mutated tumors. We also used rigosertib, despite the absence of PLK1 mutations. Viability assays allowed us to calculate the half-maximal inhibitory concentration IC50 for each drug (Fig. S4A and Supplementary Table S6). Interestingly, the IC50 of 7T_PDO treated with adagrasib was significantly higher than that of KRAS *p.G12C*-mutant lung cancer cell lines, at 39.21 µM vs. 0.8954 µM, respectively (Fig. S4G). A similar trend was observed with rigosertib: the IC50 for 7T_PDO was notably higher compared to the 6T_PDO, which harbors a PLK1 mutation, at 76.37 µM versus 3.365 µM, respectively (Fig. S4A). Likewise, the 10T_PDO sample is characterized by mutations in PIK3CA *(p.R88Q* and *p.R108H*), ERBB2 (*p.L755S*), and PARP4 (*p.D265N*). *p.R88Q* and *p.R108H* are among the most prevalent hotspot mutations in the PIK3CA gene associated with EC [29]. These mutations have been identified across various subtypes, including endometrioid adenocarcinoma, serous adenocarcinoma, undifferentiated carcinoma, and mixed epithelial carcinoma or adenocarcinoma [29]. Preclinical studies have shown that both mutations confer a gain of function to the PIK3CA protein, promoting increased cell proliferation and migration through enhanced AKT phosphorylation [30]. Moreover, several case reports and clinical studies indicate that alpelisib is well tolerated and demonstrates activity in patients with advanced gynecologic cancers harboring activating mutations in PIK3CA (*NCT01219699*, *NCT04085653*). Given these findings, we treated the 10T_PDO sample with PIK3 inhibitors, alpelisib and gedatolisib. We found a significant reduction in both organoid viability and size following treatment with 0.1 µM of alpelisib and gedatolisib, compared to 100 µM of carboplatin plus 100 nM of paclitaxel (Fig. 4I–N, S4H and S4I and Supplementary Table S6). The *p.L755S* missense variant in the ERBB2 gene is a well-documented hotspot mutation known to activate the ERBB2 kinase. This variant has been observed across various cancer types, including breast, colon, lung, and endometrial cancers [31], with tumor-dependent responses noted. Importantly, the *p.L755S* mutation has been linked to resistance against trastuzumab, an anti-HER2 therapy, in breast cancer [32]. Consequently, our model was treated with trastuzumab deruxtecan, a HER2-directed antibody-drug conjugate that is linked to a topoisomerase inhibitor and associated with poor sensitivity. This is highlighted by the difference in the IC50 values of the 10T_PDO after anti-ERBB2 treatment (9.56 µM) compared to the plasmatic concentration (0.8 µM) (Fig. S4A, S4B). In contrast, the functional impact of the *p.D265N* mutation in the PARP4 gene remains uncertain and requires further investigation. The PARP inhibitor olaparib, which is generally well-tolerated, did not show a significant effect at a 10 µM dose. However, the IC50 value of the 10T_PDO treated with olaparib corresponds to the plasma concentration values reported in the literature (Fig. S4A, S4B). In aggregate, our findings show that EC-PDOs platform represents a powerful preclinical tool for testing drug sensitivity to targeted therapies. It also allows comparing standard versus targeted therapies which may contribute to guide personalized precision oncology.

### Characterization of cancer-associated fibroblasts in EC-PDOs

**Fig. 5:**
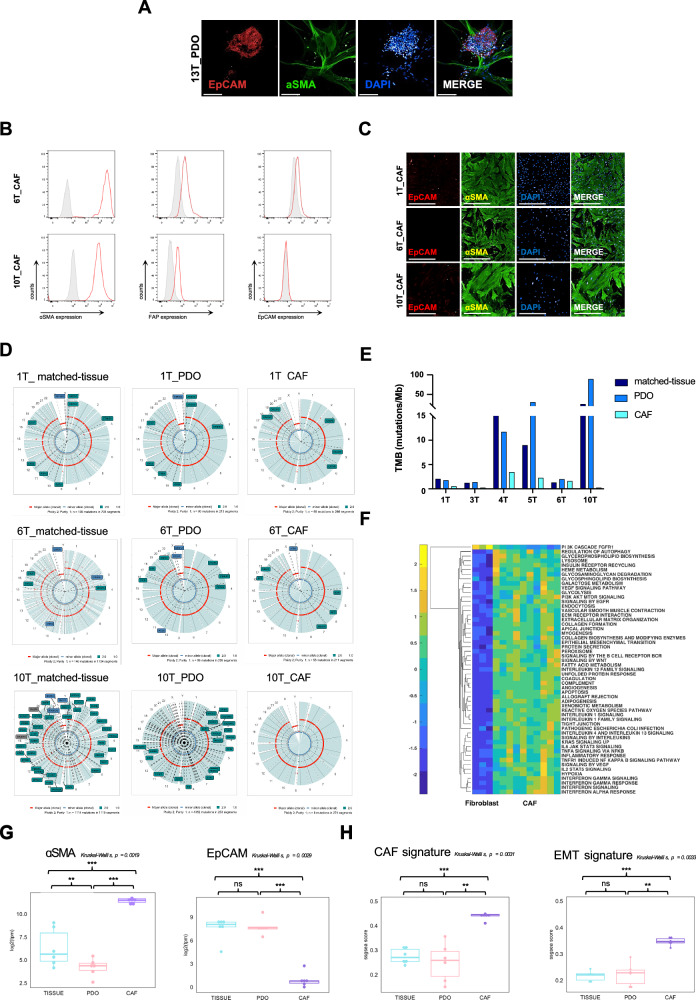
Characterization of Cancer-Associated Fibroblasts in EC PDOs. **A** Immunofluorescence assay. Representative images of 13T_PDO immunostained with anti-EpCAM (red) and anti-αSMA (green). Nuclei were stained with DAPI. Scale-bar: 200 µm. **B** Flow cytometry analysis of αSMA, fibroblast activation protein (FAP), epithelial cell adhesion molecule (EpCAM) and control Ig expression in Cancer-associated fibroblasts (CAFs) samples. **C** Immunofluorescence assay. Representative images of 1T_CAF, 6T_CAF and 10T_CAF immunostained with anti-EpCAM (red) and anti-αSMA (green). Nuclei were stained with DAPI. Scale-bar: 100 µm. **D** Karyotype profiles between matched-tissue, PDO and CAF for two selected cases. **E** Tumor mutational burden between matched-tissues, PDOs and CAFs. **F** Hierarchical clustering of selected pathways from ssGSEA across fibroblast and CAF samples. Pathway modulation between subgroups was assessed using the Wilcoxon rank-sum test, with *p-values* adjusted for multiple comparisons using the Benjamini-Hochberg procedure (FDR < 0.001). **G** Box-plot of comparative αSMA and EpCAM expression between matched-tissues, PDOs and CAFs (*p < 0.05; **p < 0.001; ***p < 0.0001). **H** Box-plot of comparative CAF and epithelial-to-mesenchymal transition (EMT) signatures between matched-tissues, PDOs and CAFs (*p < 0.05; **p < 0.001; ***p < 0.0001).

The endometrial tissue consists of a single layer of columnar epithelium, supported by an underlying stroma. Endometrial stromal cells play a crucial role for the tissue proliferation and remodeling, regulated by estrogen and progesterone hormones [33]. During organoid formation, we observed cells that spontaneously adopted an elongated, spindle-like morphology (Fig. 5A). These cells were isolated and cultured from PDO samples. After amplification and biobanking, the cells were characterized using flow cytometry analyses, immunofluorescence and immunohistochemistry. The analyses confirmed that the cells resembled CAFs phenotype, as they were positive for αSMA, fibroblast activation protein (FAP), and vimentin (VIM), while negative for epithelial cell adhesion molecule (EpCAM), cluster of differentiation 45 (CD45), and CD31 (Fig. 5B, C and S5A-S5C). To gain a deeper understanding of CAF populations, we performed WES and LOH analyses. Notably, our findings revealed a significant reduction in genomic alterations and LOH status, along with a lower tumor mutational burden (TMB) in CAFs compared to the matched tumor tissues and PDOs (Fig. 5D, E, S5D and S5E supplementary table S7). Specifically, CAFs exhibited a TMB range of 0.24 to 3.47 mutations/Mb, reflecting their relatively low heterogeneity and mutational rate.

To further characterize the isolated CAFs, we conducted bulk RNA sequencing analysis. Specifically, we performed single-sample gene set enrichment analysis (ssGSEA) on the CAF samples and compared them to endometrial stromal fibroblast samples obtained from the GEO database (ID: GSE114296). A total of 1590 pathways from the HALLMARK, REACTOME, and KEGG databases were analyzed to assess differences in pathway score profiles between CAF and fibroblast samples (Fig. S6A). The results revealed that CAFs are markedly distinct from fibroblasts, exhibiting significant enrichment in pathways associated with metabolism, immune responses, and cancer-related processes (Fig. 5F). Notably, key CAF markers, including αSMA, VIM, FAP, and CD90, along with CAF and epithelial-to-mesenchymal transition (EMT) signatures, were upregulated in CAFs relative to matched-tissues and matched-PDOs. Conversely, the epithelial marker EpCAM and CD45 were downregulated in CAFs (Fig. 5G, H and S6B). Taken together, these findings confirm that CAFs, which were isolated and thoroughly characterized using multiple assays, are a prominent component of EC_PDOs.

### EC-CAFs exhibit poorer response to standard chemotherapy than matched EC-PDOs

**Fig. 6:**
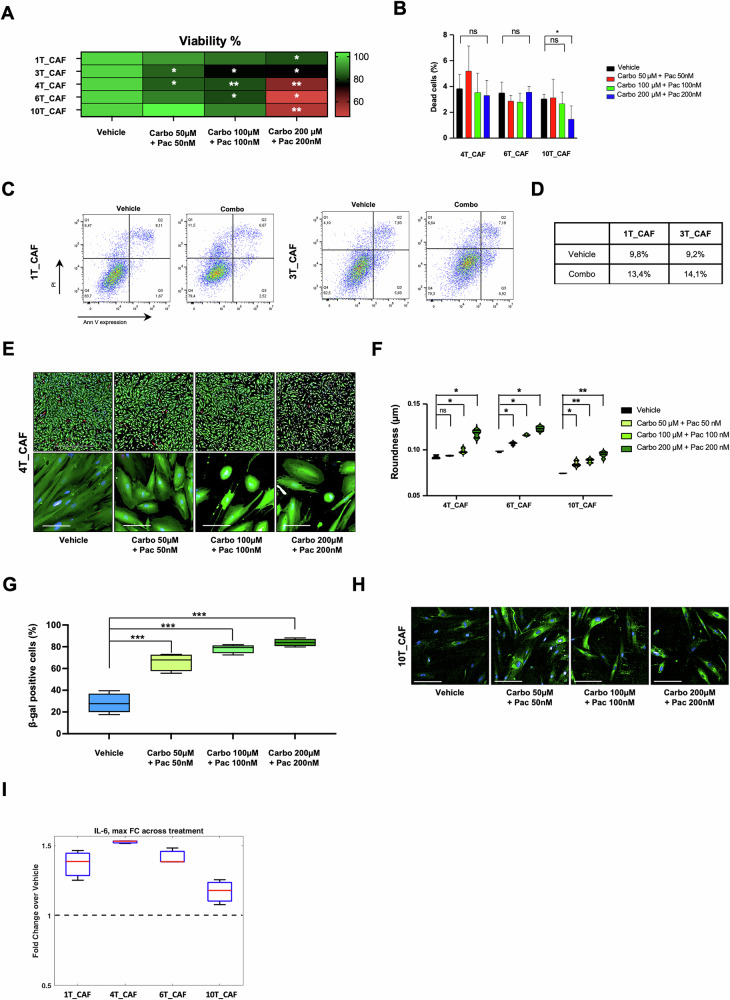
CAFs response to standard chemotherapy. **A** Heatmap of CAFs viability following carboplatin + paclitaxel (50 µM + 50 nM; 100 µM + 100 nM; 200 µM + 200 nM) treatment for 72 h, evaluated by ATPlite luminescence assay. The *p-value* is calculated from three independent experiments (*p < 0.05; **p < 0.001; ***p < 0.0001). **B** Percentage of dead cells from three independent experiments following carboplatin + paclitaxel (50 µM + 50 nM; 100 µM + 100 nM; 200 µM + 200 nM) treatment for 72 h using live-dead cell viability assay kit assessed by Opera Phenix Plus High-Content Screening System (*p < 0.05; **p < 0.001; ***p < 0.0001). **C** Flow cytometry analysis of CAFs following carboplatin (100 µM) + paclitaxel (100 nM) treatment for 72 h. Cytotoxic effects were shown as percentage of PI and AnnV^+^ cells. **D** Percentage of PI and AnnV^+^ positive dead cells following carboplatin (100 µM) + paclitaxel ^(^100 nM) treatment for 72 h assessed by flow cytometry analysis. **E** Representative immunofluorescence images showing the morphological differences of 4T_CAF following carboplatin + paclitaxel (50 µM + 50 nM; 100 µM + 100 nM; 200 µM + 200 nM) treatment for 72 h using live-dead cell viability assay kit assessed by Opera Phenix Plus High-Content Screening System. Scale-bar: 2 mm (up) and 50 µm (down). **F** Violin plot of CAFs roundness following carboplatin + paclitaxel (50 µM + 50 nM; 100 µM + 100 nM; 200 µM + 200 nM) treatment for 72 h using live-dead cell viability assay kit assessed by Opera Phenix Plus High-Content Screening System. Box-plot (**G**) and representative immunofluorescence images (**H**) of beta-galactosidase positive 10T_CAF following carboplatin + paclitaxel (50 µM + 50 nM; 100 µM + 100 nM; 200 µM + 200 nM) treatment for 72 h using cell event senescence green detection kit assessed by Opera Phenix Plus High-Content Screening System. The *p-value* is calculated on 9 fields of each well (*p < 0.05; **p < 0.001; ***p < 0.0001). Scale-bar: 50 µm. **I** Box-plot of maximum level of IL-6 cytokine measured in the culture media of the CAFs following carboplatin + paclitaxel (50 µM + 50 nM; 100 µM + 100 nM; 200 µM + 200 nM) treatment for 72 h.

CAFs have been extensively shown to support cancer development and progression, as well as influence treatment response. In this study, we investigated the effects of standard EC chemotherapy on CAFs compared to their matched EC-PDOs. Following their isolation and characterization, CAFs from 1 T, 3 T, 4 T, 6 T and 10 T samples were treated with carboplatin plus paclitaxel at different concentrations (50 µM + 50 nM, 100 µM + 100 nM, 200 µM + 200 nM) for 72 hours. As shown in Fig. 6A–D, treated CAFs exhibited a poorer response to the combination treatment than PDOs, as assessed by several assays and instruments. The viability of CAFs was measured by ATPlite luminescence assay (Fig. 6A). Additionally, CAFs response was evaluated through the Live-Dead Cell Viability assay, which assesses membrane integrity and esterase activity, by the Opera Phenix Plus High-Content Screening System and FACS analysis of Annexin V and PI staining (Fig. 6B–D, S7A and S7B, respectively). Interestingly, we observed significant morphological changes in treated CAFs (Fig. 6E, F, S7C and S7D). These changes included a reduction in cells area and an increase in cell roundness suggesting a notable impact of the treatment on CAF morphology. Given the resistance of CAFs to standard chemotherapy, we hypothesized that senescence might be a contributing factor to this event. To explore this possibility, we conducted a senescence assay using β-galactosidase hydrolysis, a widely recognized biomarker for senescent cells. As shown in Fig. 6G, H, S7E and S7F, chemotherapy-treated CAFs exhibited a marked increase in β-galactosidase positivity, which rose in a dose-dependent manner with increasing drug concentrations. Moreover, it is known that senescent cells release interleukins, pro-inflammatory cytokines, and growth factors that influence the behavior and function of neighboring cells [34]. Based on this, we evaluated the levels of IL-6 in culture media samples following chemotherapy treatment. As shown in Fig. 6I, Il-6 protein levels were elevated in all our samples.

Additionally, increased nuclear expression of the cyclin-dependent kinase (CDK) inhibitors 1 and 2 A (commonly known as p21 and p16^INK4a^) and p53 [35], as assessed by immunofluorescence, suggests a potential induction of cellular senescence following treatment (Fig. S7G and S7H). Collectively, these data indicate that endometrial CAFs, are resistant and undergo senescence upon standard chemotherapy treatment.

### PDOs predict clinical outcomes of EC patients treated with adjuvant chemotherapy

**Fig. 7:**
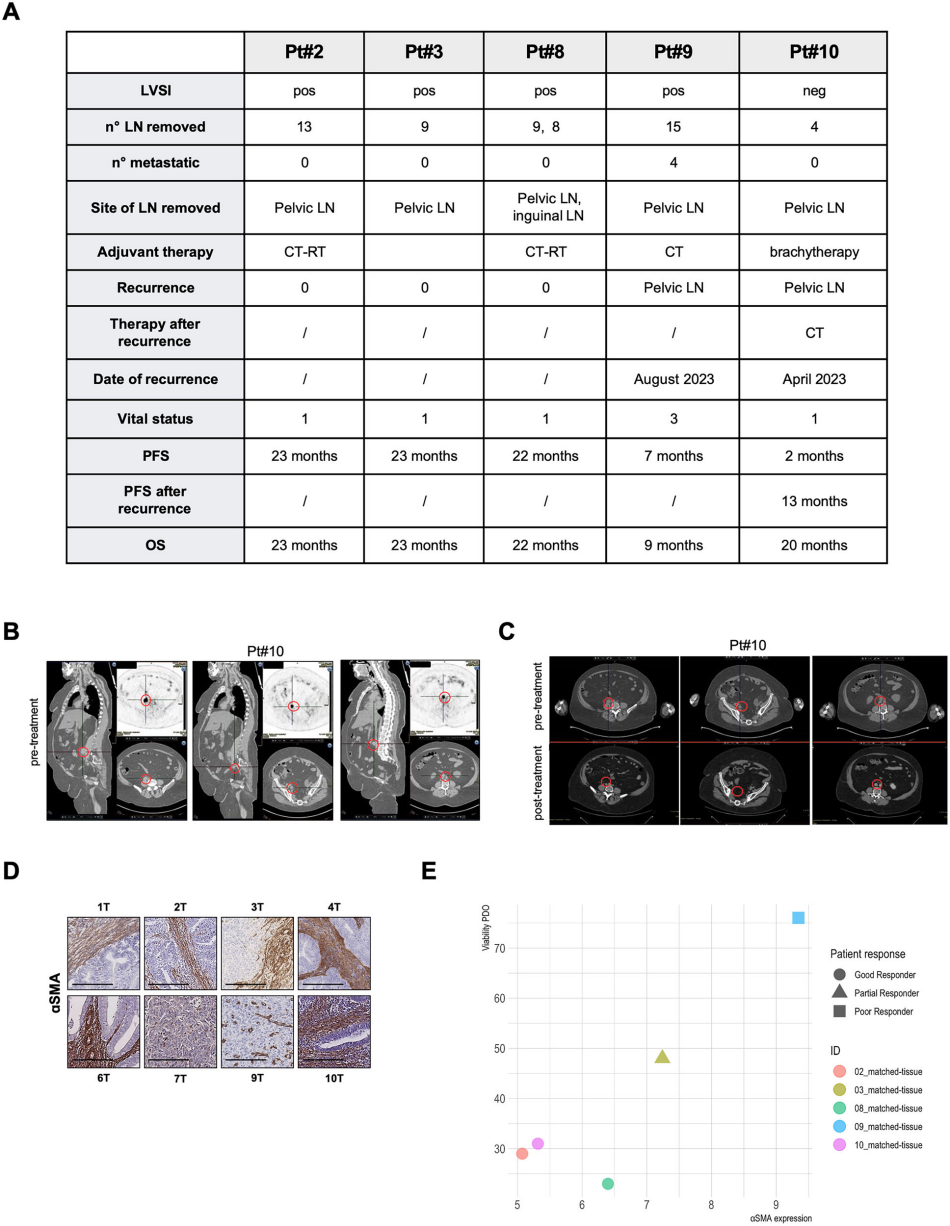
PDOs predict clinical outcomes of EC patients treated with adjuvant chemotherapy*.* **A** Clinical information about patients’ follow-up. LVSI (LymphoVascular Space Involvement) (0= no; 1= yes (massive); 2= focal); LN (lymphonode); CT (chemotherapy); RT (radiotherapy); vital status (1= alive without evidence of disease; 2= alive with tumor; 3= died with disease; 4= died of other cause(s) with evidence of disease; 5= died of other cause(s) without evidence of disease); PFS (progression free survival); OS (overall survival). **B** Tumor CT scans images of three different metastatic site of Pt#10 after 2 months from surgery. **C** Tumor CT scans images of Pt#10 pre (12nd June 2023) and after (13rd September 2024) adjuvant treatment. **D** Histological analysis of alpha smooth muscle antigen (αSMA) in EC tumors. Scale-bar: 200 µm. **E** Scatter plot of relative αSMA expression of each tumor samples versus PDOs viability following carboplatin (100 µM) + paclitaxel (100 nM) treatment for 72 h and patient response.

Once proven that organoids faithfully recapitulate tumor profiling, to determine whether our PDOs can serve as a valid model for predicting patient responses to treatment, we compared the viability of PDOs with patient’s oncological outcomes. Among 10 patients studied, 5 were classified as high risk based on their clinico-histologic and biomolecular characteristics and underwent a complete surgical cytoreduction (Fig. 7A). On these, patients #2, #3, #8, and #10 exhibited a good response to therapy, while patient #9 showed a poor response. Correspondingly, the viability of each patient’s PDOs after standard chemotherapy was classified as good or poor based on their observed treatment responses. Notably, all five PDOs mirrored the patients’ responses to therapy. In details: Pt#2 after surgical cytoreduction and standard chemotherapy treatment has no recurrences with an OS ad PFS of 23 months; Pt#8 after surgical cytoreduction and standard chemotherapy treatment has no recurrences with an OS ad PFS of 22 months (Fig. S8A); Pt#10 after 2 months post-surgery was diagnosed to have a pelvic lymph nodes recurrence (Fig. 7B), she underwent standard therapy by having a complete response with an OS of 20 and a subsequent PFS of 13 months (Fig. 7C); Pt#3 underwent chemotherapy, but it was not completed due to toxicity: she had no recurrences with an OS and PFS of 23 months (Fig. S8B); Pt#9 after optimal surgical cytoreduction, as shown by post-surgical CT scan (Fig. S8C), and subsequent adjuvant chemotherapy treatment interrupted for toxicity, she showed a disease’s progression, with a PFS of 7 months and an OS of 9 months. Considering the role of CAFs and their previously demonstrated resistance to chemotherapy, we next investigated whether the presence of CAFs in primary tumors, assessed by αSMA expression levels (Fig. 7D), could be correlated with PDOs viability and, if possible, patient’s follow-up (Figs. 7E and S8D). Specifically, in tumors with low αSMA expression and high EpCAM expression, such those from Pt#2, Pt#8 and Pt#10, a strong response to treatment was observed. In contrast, the tumor from Pt#9 tumor, characterized by high αSMA and low EpCAM expression, demonstrated poor treatment responsiveness. Notably, the Pt#3 tumor, despite having high EpCAM expression and high αSMA expression compared to samples Pt#2, Pt#8 and Pt#10, exhibited partial treatment response. Furthermore, our findings show that EC-PDOs hold the potential to predict the chemosensitivity of carboplatin and taxol and the clinical prognosis of patients. Although more prospective multicenter data are needed to validate our findings, we show that chemosensitivity measured with 3D pre-clinical models such as organoids might be used as a predictive tool to identify the risk of disease progression in EC patients.

## Discussion

There is an unprecedent need to generate tools to predict patient response to standard and targeted therapies. These specific tools need to be faithfully surrogates of cancer patients thus intercepting patient heterogeneity and complexity. They also need to be generated in a time frame compatible with the administration of a given cancer treatment. In the present study, we provide proof of concept that primary endometrial cancer (EC) tissues grown as organoid cultures faithfully model the genetic complexity, the tumor microenvironment and the drug response of human EC in vitro. We found that EC-PDOs recapitulate patient response to standard therapy. These findings might pave the way of using PDOs as a robust EC patient surrogate for predicting the response to standard therapy. The possibility to compare the efficacy of standard therapy versus targeted approaches for those EC patients carrying specific druggable alterations is certainly one of the most important advantages of PDOs. Indeed, we assessed the efficacy of PI3K inhibitors that have been recently used in clinical trials for advanced or metastatic EC, including gedatolisib (*NCT01420081, NCT02069158*) and buparlisib (*NCT01289041, NCT01397877*). Our findings clearly show that PI3K inhibitors could be used either as a first-line therapy or represent a second line for EC patients developing resistance to standard therapy and carrying druggable PI3K mutations. We also provide evidence that EC-PDOs platform faithful and precisely resembles the response to the specific targeted therapies as for 7T_PDO sample, which carries KRAS *p.G12D* mutation. Interestingly, the treatment with adagrasib, a selective inhibitor of KRAS *p*.G12C was ineffective on 7T_PDO sample (Fig. S4A). Growing evidence have clearly shown the critical role of tumor microenvironment on the prognosis and the response to therapy of cancer patients. TME is a complex mixture of stromal, vascular and immune cells whose respective percentage varies across different type of tumors. Cancer progression is frequently driven by dynamic interactions between tumor cells and the surrounding stromal cells, especially with CAFs. CAFs are found in both primary and metastatic tumors, including those of pancreas, breast, colon, liver and endometrium, and are known to contribute to the development of drug resistance across various solid tumors [36–39]. Indeed, we found in primary EC tumors that alpha smooth muscle antigen (αSMA)-positive cells represent a significant component of endometrial tissue. It is known that stromal cells play a pivotal role in regulating the development, growth, differentiation, and function of the overlying endometrium cells in adult tissue [40]. Additionally, their role in oncology is intensively studied as they are involved in tumor key process such as progression, recurrence, metastasis and resistance to therapy [41, 42]. Recent preclinical and translational research has highlighted the diverse roles of CAFs in promoting tumor phenotypes that correlate with poor clinical outcomes [43, 44]. We found that EC_CAFs had a poorer response to standard treatment than matched-PDOs. Interestingly, chemotherapy treated CAFs undergo senescence by reshaping TME through paracrine networks toward resistance and progression disease. 3D modeling of tumor cells and surrounding tumor microenvironment cellular landscape is intensively studied but it is still not applicable for clinical purpose. While organoids, at very early passages, may also recapitulate the complex cellular composition of the tumor and surrounding tumor microenvironment, additional 3D ex vivo approaches could be potentially more representative than PDOs when looking at primary tumors. Undoubtedly, 3D preclinical tools will play a crucial role to instruct innovative therapeutic regiments aimed at overriding resistance developed upon first- and second-line treatments. Biobanking of PDOs derived from primary tumors and from matched resistant cancer tissues as they evolve under the selective pressure of specific anticancer treatment might represent a unique and unprecedently valuable preclinical tool to dissect mechanistically and override resistance. As interventional trials using PDOs either alone or in co-culture with TME components will be employed and related data will be available, we could realistically evaluate how broad the impact of 3D pre-clinical models will be on cancer therapy.

## Supplementary information

Supplementary Figures

Supplementary Tables

Supplementary Legends

## Data Availability

Any information required to reanalyze the data reported in this paper is available from the lead contact upon request.

## Code availability

Any information required to reanalyze the data reported in this paper is available from the lead contact upon request.

## Materials availability

Materials generated in this study will be available upon fulfillment of the material transfer agreement (MTA).
