# Supplementary material for: Dissecting endometrial cancer complexity in response to standard and targeted therapies
Source: Cell Death Dis. 2025 Nov 28;16(1):873. doi: 10.1038/s41419-025-08051-8 (PMC12663567; doi:10.1038/s41419-025-08051-8)

Supplementary Fig. 1

A

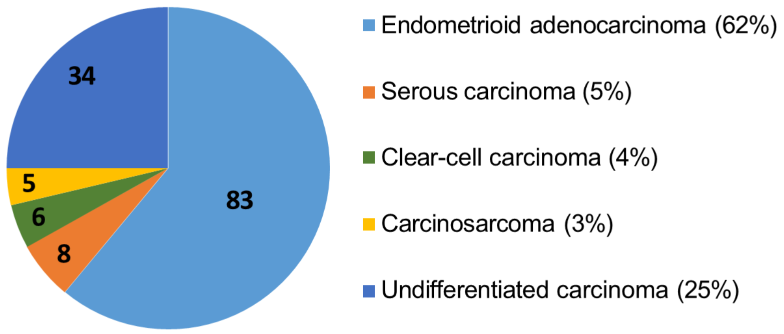

B

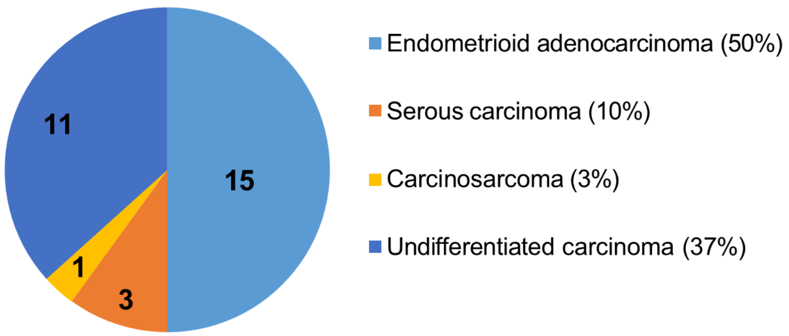

Supplementary Fig. 2

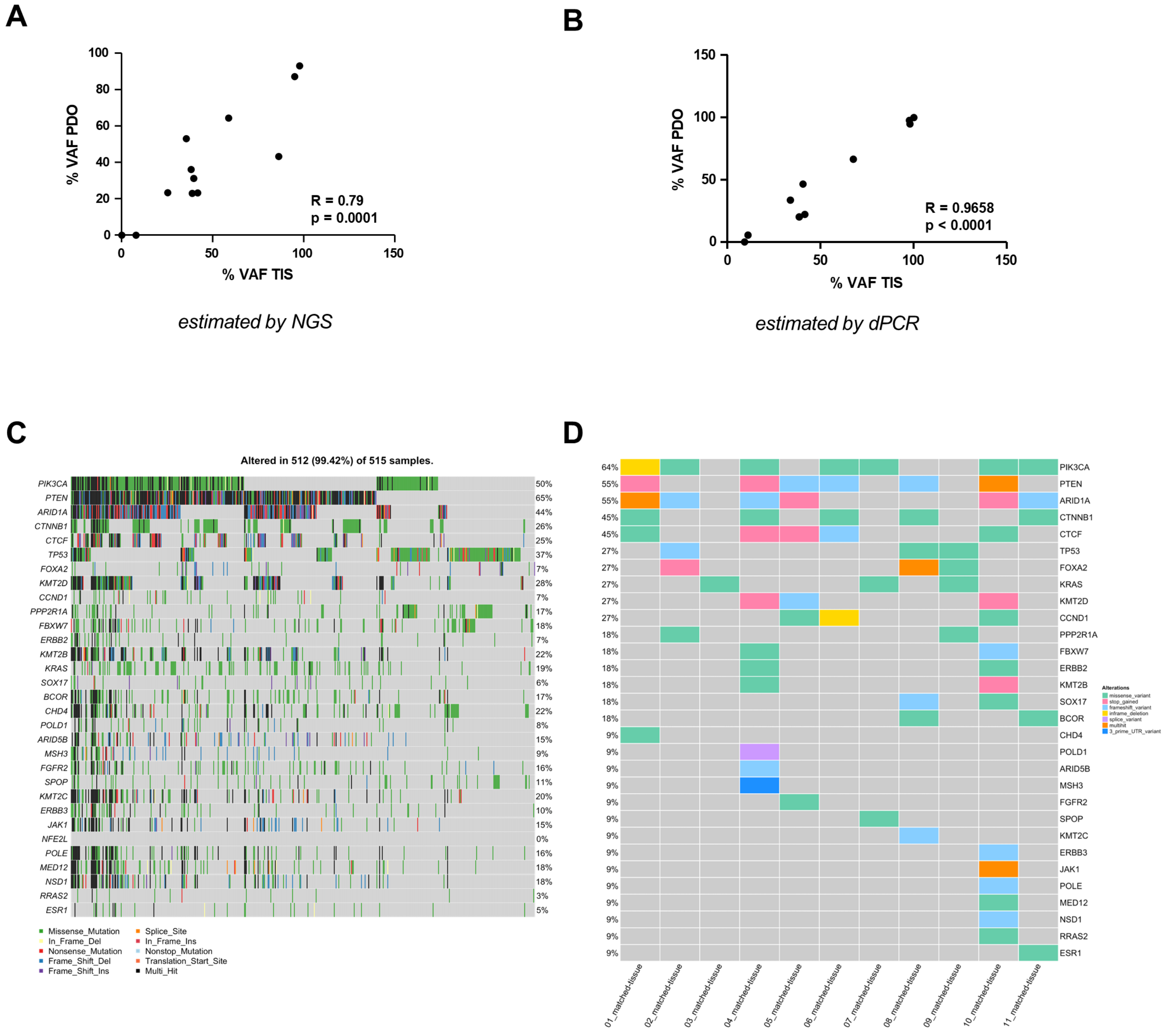

Supplementary Fig. 3

A

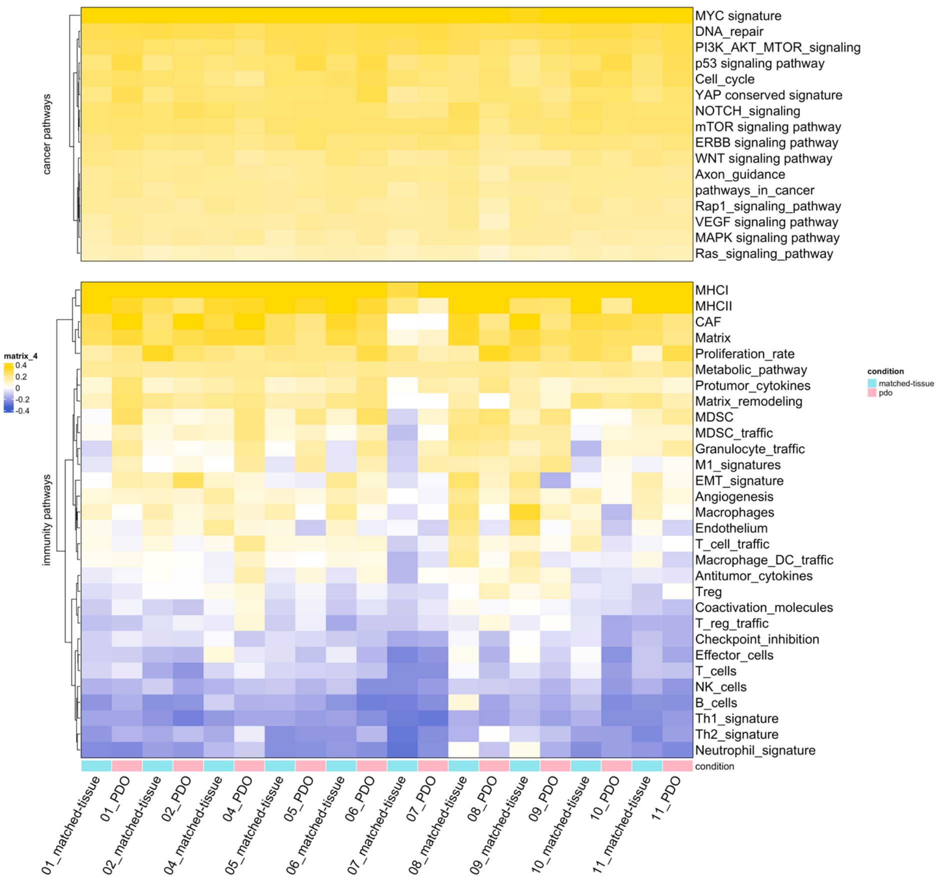

B

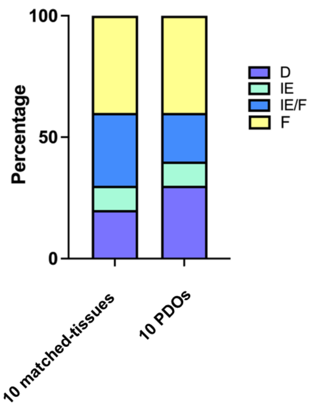

B

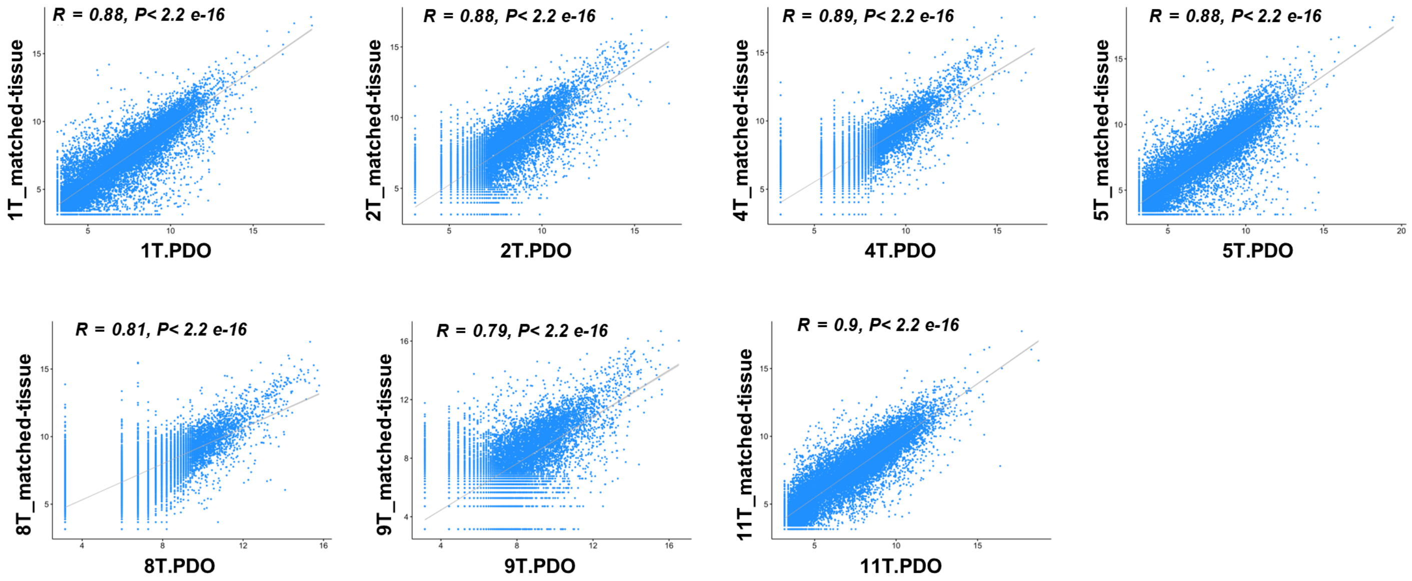

C

DRUGS	Plasmatic Concentration (µM)
Carboplatin	188
Paclitaxel	0,228

D

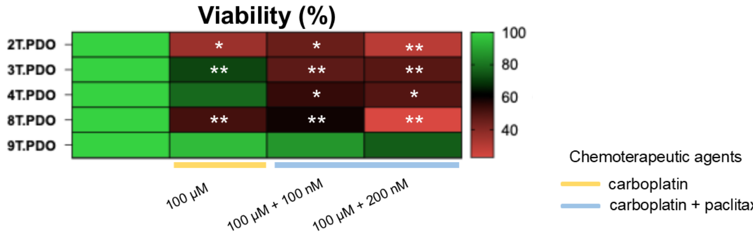

Supplementary Fig. 4

A

	IC50 (µM)		
**DRUGS**	**6T_PDO**	**7T_PDO**	**10T.PDO**
**Adagrasib**	*n.a.*	39,21	*n.a.*
**Alpelisib**	1,391	6,811	0,17
**Buparlisib**	*n.a.*	2,767	*n.a.*
**Gedatolisib**	0,1321	0,4216	0,0033
**Ipatasertib**	*n.a.*	9,97	*n.a.*
**Olaparib**	*n.a.*	*n.a.*	10,52
**Rigosertib**	3,365	76,37	*n.a.*
**Trastuzumab Deruxtecan**	*n.a.*	*n.a.*	9,56
**Volasertib**	3,415	*n.a.*	*n.a.*

B

**DRUGS**	**Plasmatic concentration (µM)**
**Adagrasib**	1,63
**Alpelisib**	4,91
**Buparlisib**	2,8
**Gedatolisib**	5,8
**Ipa tasertib**	2,1
**Olaparib**	17,5
**Rigosertib**	14,5
**Trastuzumab Deruxtecan**	0,8
**Volasertib**	0,92

C

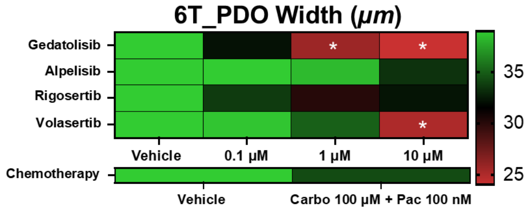

D

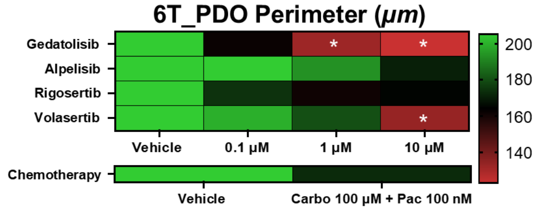

E

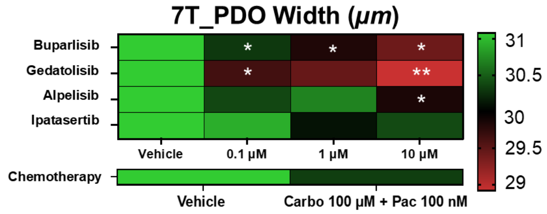

F

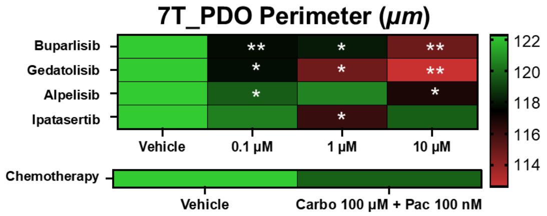

G

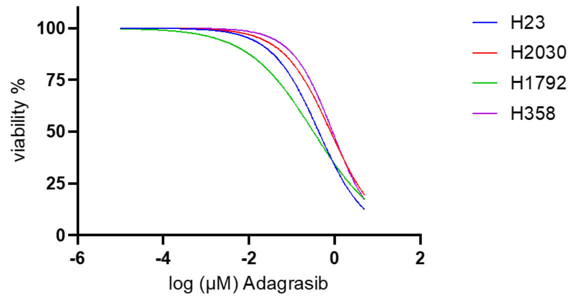

**ADAGRASIB**	**IC50 (µM)**
**H23**	0,4265
**H2030**	0,8075
**H1792**	0,3190
**H358**	0,8954

H

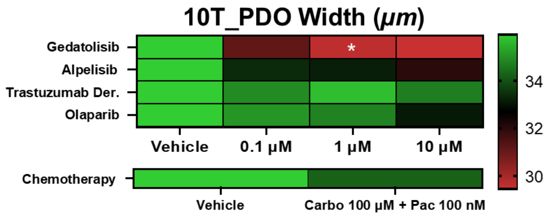

I

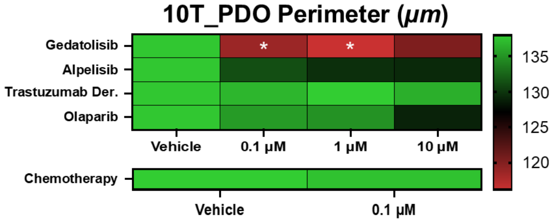

Supplementary Fig. 5

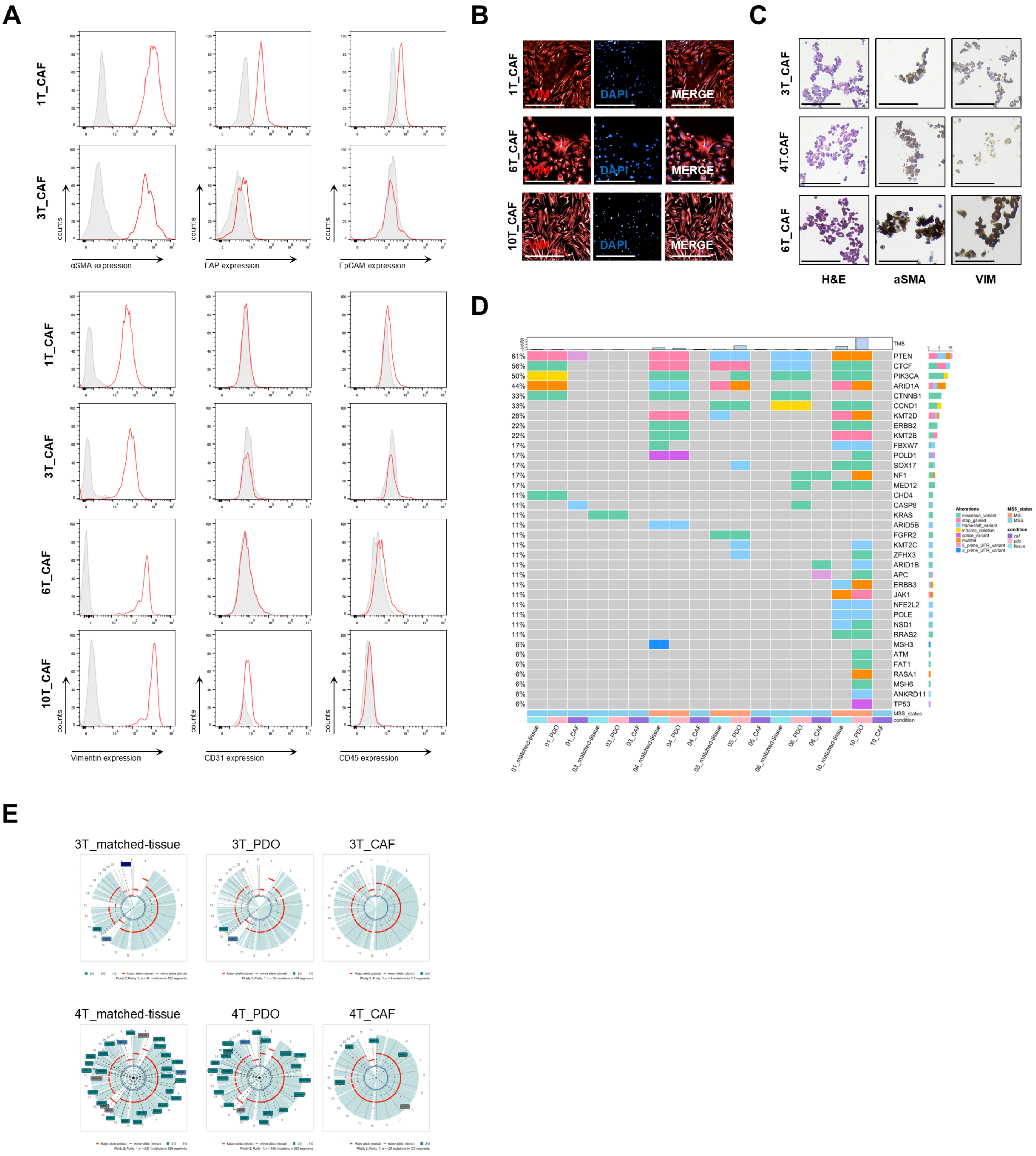

Supplementary Fig. 6

A

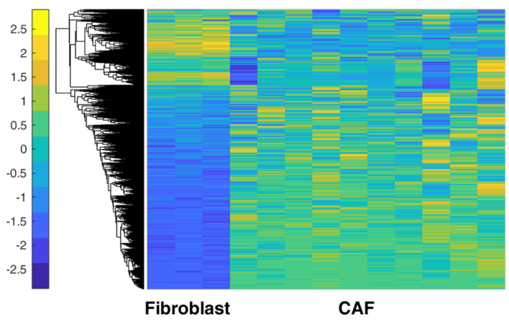

B

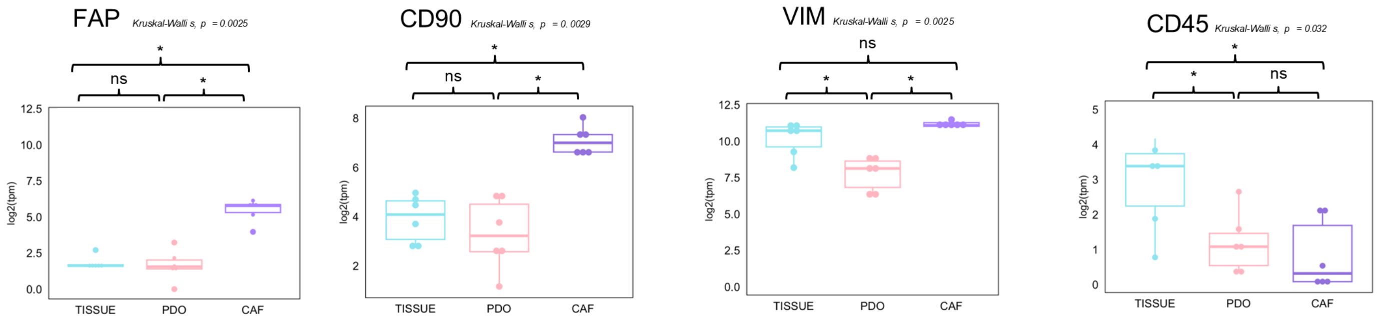

Supplementary Fig. 7

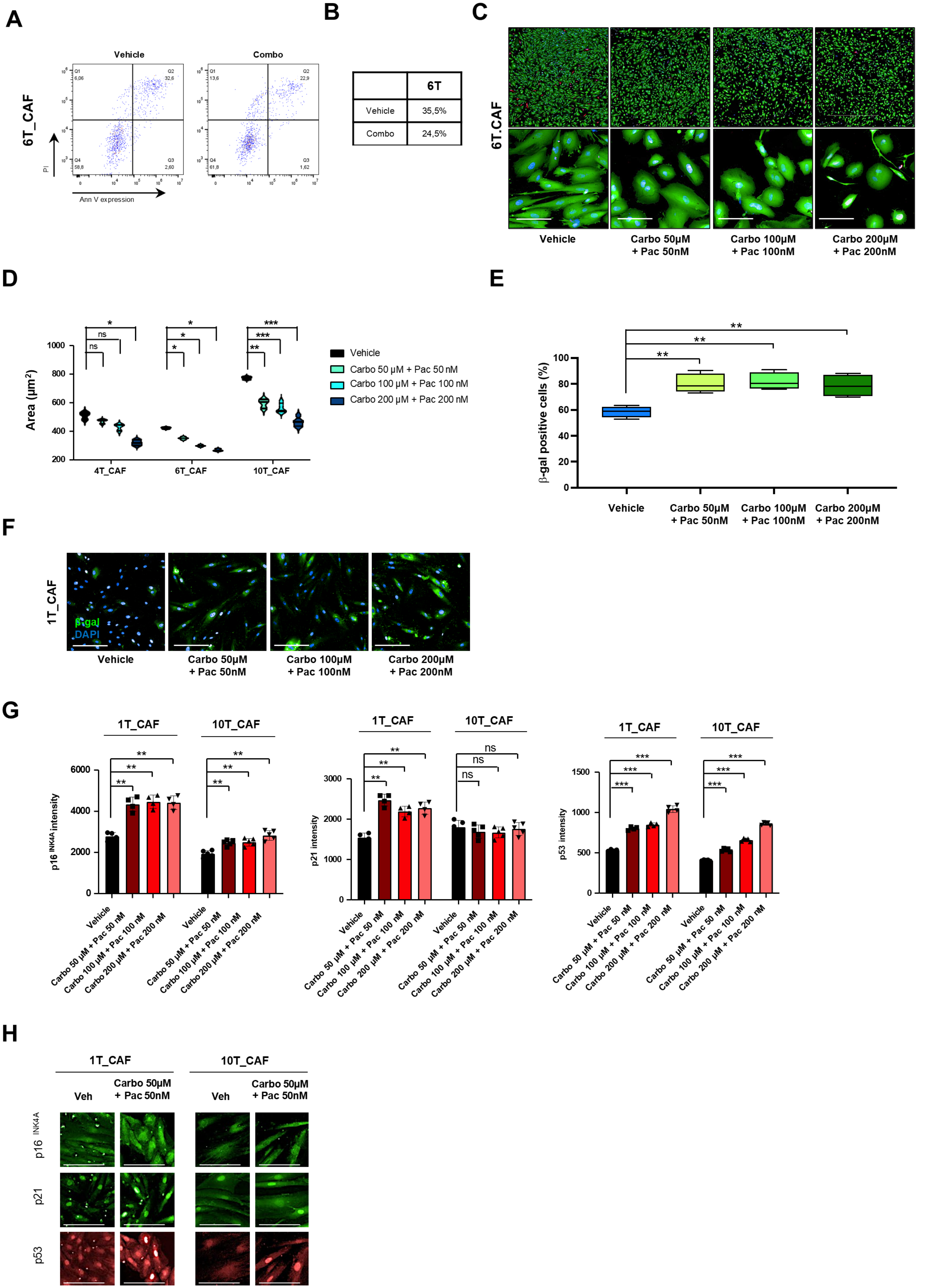

Supplementary Fig. 8

A

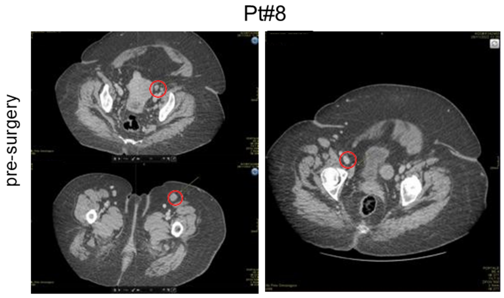

B

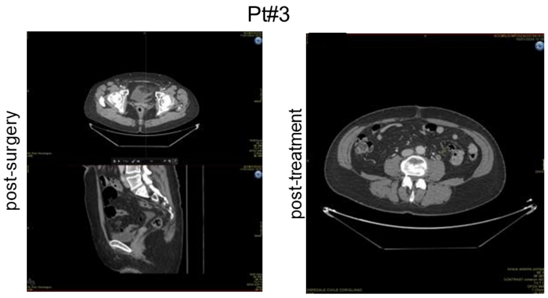

C

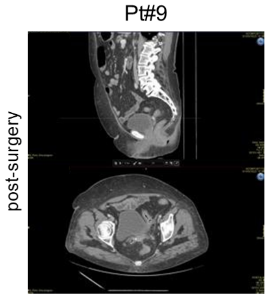

D

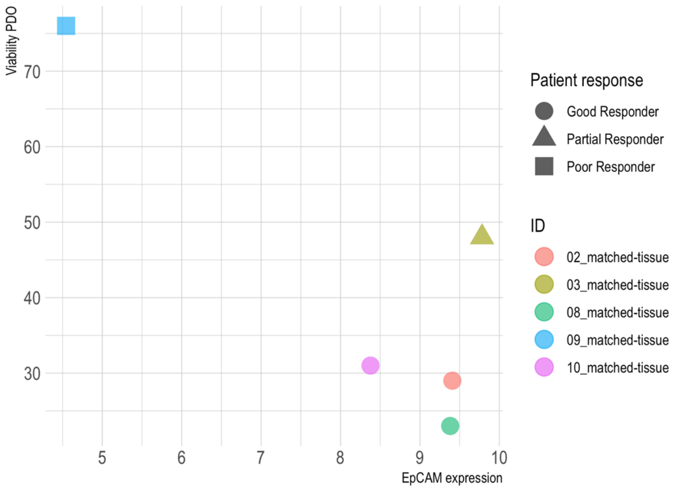

Supplement: Supplementary file 1 — Supplementary Figures [file 41419_2025_8051_MOESM1_ESM.pdf]
