# Supplementary material for: Dissecting endometrial cancer complexity in response to standard and targeted therapies
Source: Cell Death Dis. 2025 Nov 28;16(1):873. doi: 10.1038/s41419-025-08051-8 (PMC12663567; doi:10.1038/s41419-025-08051-8)
Supplement: Supplementary file 3 — Supplementary Legends [file 41419_2025_8051_MOESM3_ESM.pdf]

Supplementary Figure 1. (**A**, **B**) Pie charts comparing the stratification of all tumours based on histological type (A) with that of all derived organoids (B).

Supplementary Figure 2. (**A**, **B**) **Pearson correlation analysis** of comparison of VAF of genetic alterations detected in PDOs and their matched tumours, estimated by NGS (A) and digital PCR (B). (**C, D**) **Heatmaps** of most frequently gene mutations in TCGA cohort (C) and our biobank (D) respectively.

Supplementary Figure 3. (**A**) **ssGSEA pathways heatmap** of cancer (up) and immunity (down) expression between parental tumor and corresponding PDO. (**B**) **Deconvolution analysis** of 10 samples in four different immunosubtype immune-enriched fibrotic (IE/F), immune-enriched (IE), fibrotic (F) and depleted (D). (**C**) **Pearson correlation analysis** between matched-tissues and PDOs. (**D**) List of **plasmatic concentration** of carboplatin and paclitaxel. (**E**) **Heatmap** of PDOs viability following carboplatin (100 μM), carboplatin + paclitaxel (100 μM + 100 nM; 100 μM + 200 nM) treatment for 72h, evaluated by ATPlite luminescence assay. The SD and *p-value* is calculated from three independent experiments (* $p< 0.05$; ** $p< 0.001$; *** $p< 0.0001$).

Supplementary Figure 4. (**A**) List of **IC50 value** of dose response after 72h of treatment. (**B**) List of **plasmatic concentration** of molecular drugs. (**C, E, H**) **Heatmaps** of 6T_PDO (C), 7T_PDO (E) and 10T_PDO (H) width following gedatolisib (0.1-1-10 μM), alpelisib (0.1-1-10 μM), rigosertib (0.1-1-10 μM), volasertib (0.1-1-10 μM), buparlisib (0.1-1-10 μM), ipatasertib (0.1-1-10 μM), adagrasib (0.1-1-10 μM), olaparib (0.1-1-10 μM), trastuzumab deruxtecan (0.1-1-10 μM) and carboplatin (100 μM) + paclitaxel (100 nM) treatment for 72h, evaluated by Opera Phenix Plus high throughput microplate confocal imager. The *p-value* is calculated from three independent experiments (* $p< 0.05$; ** $p< 0.001$; *** $p< 0.0001$). (**D, F, I**) **Heatmaps** of 6T_PDO (D), 7T_PDO (F) and 10T_PDO (I) perimeter following gedatolisib (0.1-1-10 μM), alpelisib (0.1-1-10 μM), rigosertib (0.1-1-10 μM), volasertib (0.1-1-10 μM), buparlisib (0.1-1-10 μM), ipatasertib (0.1-1-10 μM), adagrasib (0.1-1-10 μM), olaparib (0.1-1-10 μM), trastuzumab deruxtecan (0.1-1-10 μM) and carboplatin (100 μM) + paclitaxel (100 nM) treatment for 72h, evaluated by Opera Phenix Plus high throughput microplate confocal imager. The *p-value* is calculated from three independent experiments (* $p< 0.05$; ** $p< 0.001$; *** $p< 0.0001$). (**G**) **Representative dose-response curve** for adagrasib evaluated by ATPlite luminescence assay.

Supplementary Figure 5. (**A**) **Flow cytometry analysis** of αSMA, FAP, vimentin, EpCAM, CD31, CD45 and control Ig expression in CAFs samples. (**B**) **Immunofluorescent assay.** Representative images of 1T_CAF, 6T_CAF and 10T_CAF immunostained with anti-vimentin (red). Nuclei were stained with DAPI. Scale-bar: 100 μm. (**C**) **Histological analysis** of alpha smooth muscle antigen (αSMA) and vimentin (VIM) of 3T_CAF, 4T_CAF and 6T_CAF. Scale-bar: 100 μm. (**D**) **Heatmap** of gene mutation variations between parental tumour, corresponding PDO and matched CAFs in the most frequently mutated genes of endometrial cancer. Microsatellites status and TMB are reported on the bottom and on the top, respectively. (**E**) **Karyotype profiles** between matched-tissue, PDO and CAF for two selected cases.

Supplementary Figure 6. (**A**) **Hierarchical clustering** of 1590 pathway scores obtained from single-sample gene set enrichment analysis (ssGSEA). For each pathway, the scores were standardized between fibroblast and CAF samples. (**B**) **Box-plots** of comparative FAP, CD90, VIM and CD45 expression between matched-tissues, PDOs and CAFs (* $p < 0.05$; ** $p < 0.001$; *** $p < 0.0001$).

Supplementary Figure 7. (**A**) **Flow cytometry analysis** of CAFs following carboplatin (100 µM) + paclitaxel (100 nM) treatment for 72h. Cytotoxic effects were shown as percentage of PI and $AnnV^{+}$ cells. (**B**) **Percentage** of PI and $AnnV^{+}$ positive dead cells following carboplatin (100 µM) + paclitaxel (100 nM) treatment for 72h assessed by flow cytometry analysis. (**C**) **Representative immunofluorescence images** showing the morphological differences of 6T_CAF following carboplatin + paclitaxel (50 µM + 50 nM; 100 µM + 100 nM; 200 µM + 200 nM) treatment for 72h using live-dead cell viability assay kit assessed by Opera Phenix Plus High-Content Screening System. Scale-bar: 2 mm (up) and 50 µm (down). (**D**) **Violin plot** of CAFs area following carboplatin + paclitaxel (50 µM + 50 nM; 100 µM + 100 nM; 200 µM + 200 nM) treatment for 72h using live-dead cell viability assay kit assessed by Opera Phenix Plus High-Content Screening System. (**E, F**) **Box-plots** (E) and **representative immunofluorescence images** (F) of beta-galactosidase positive 1T_CAF following carboplatin + paclitaxel (50 µM + 50 nM; 100 µM + 100 nM; 200 µM + 200 nM) treatment for 72h using cellevent senescence green detection kit assessed by Opera Phenix Plus High-Content Screening System. The *p-value* is calculated on 9 fields of each well (* $p < 0.05$; ** $p < 0.001$; *** $p < 0.0001$). Scale-bar: 50 µm. (**G**) **Histograms** show nuclear $p16^{INK4A}$ p21 and p53 intensity in 1T_CAF and 10T_CAF following carboplatin + paclitaxel (50 µM + 50 nM; 100 µM + 100 nM; 200 µM + 200 nM) treatment for 72h assessed by Opera Phenix Plus High-Content Screening System. The *p-value* is calculated on 25 fields of each well at different planes (* $p < 0.05$; ** $p < 0.001$; *** $p < 0.0001$). (**H**) **Representative immunofluorescence images** of nuclear $p16^{INK4A}$, p21 and p53 in 1T_CAF and 10T_CAF following carboplatin (50 µM) + paclitaxel (50 nM) treatment for 72h assessed by Opera Phenix Plus High-Content Screening System. Scale-bar: 200 µm.

Supplementary Figure 8. (**A**) **Tumour CT scans** images of two pre-surgical different metastatic site of Pt#8. (**B**) **Tumour CT scans** images of Pt#3 post surgery and post adjuvant treatment. (**C**) **Tumour CT scan** image of Pt#9 post-surgery. (**D**) **Scatter plot** of relative EpCAM expression of each tumour samples versus PDOs viability following carboplatin (100 µM) + paclitaxel (100 nM) treatment for 72h and patient response.

Supplementary Table S1. ***Clinical features of enrolled patients.***

Grade (*1= grade 1; 2= grade 2; 3= grade 3*); myometrial infiltration (*1< 50%; 2>50%*); cervical stromal invasion (*0= no; 1= yes; 9= unknown*); histology (*1= endometroid;2= undifferentiated; 3= carcinosarcoma; 4= mixed (endometroid/serous)*); surgical approach (*1= minimally invasive (MIS); 2= MIS robotic; 3= open*); TP53 (*0< 50%*); MLH1, MSH2, MSH6, MSH6 + PMS2 (*0= stable; 1= high instability; 2= low instability*); POLE mut (*0= no; 1= yes; 2= VUS*).

Supplementary Table S2. ***PDOs biobank features.***

The table reports the main analyses performed on each PDO, including histological assessment, molecular profiling and standard treatment testing. For each PDOs line, the table also shows the total culture time (in months) and number of passages achieved. PDOs passages (Px), Whole Exome Sequencing (WES), RNA sequencing (RNA-seq), Immunohistochemistry (IHC), not available (n.a.).

Supplementary Table S3. ***VAF and mutated residues in matched-tissue and PDO.***

Supplementary Table S4. ***Mean and standard deviation of viability after chemotherapy treatment.***

Mean percentage and standard deviation of cell viability for 6T, 7T, and 10T PDOs after 72 hours of standard treatment assessed by ATP luminescence assay.

Supplementary Table S5. ***Number of PDOs after chemotherapy treatment.***

Mean of number of PDOs for 6T, 7T, and 10T PDOs after 72 hours of standard treatment evaluated using the Opera Phenix's machine learning.

Supplementary Table S6. ***Mean and standard deviation of viability after targeted therapy.***

Mean percentage and standard deviation of cell viability for 6T, 7T, and 10T PDOs after 72 hours of targeted treatment assessed by ATP luminescence assay.

Supplementary Table S7. ***VAF and mutated residues in matched-tissue, PDO and CAF.***
